# Genome-Wide Analysis of Codon Usage and Influencing Factors in Chikungunya Viruses

**DOI:** 10.1371/journal.pone.0090905

**Published:** 2014-03-04

**Authors:** Azeem Mehmood Butt, Izza Nasrullah, Yigang Tong

**Affiliations:** 1 Centre of Excellence in Molecular Biology (CEMB), University of the Punjab, Lahore, Pakistan; 2 Department of Biochemistry, Faculty of Biological Sciences, Quaid-i-Azam University, Islamabad, Pakistan; 3 State Key Laboratory of Pathogen and Biosecurity, Beijing Institute of Microbiology and Epidemiology, Beijing, People’s Republic of China; Fondazione IRCCS Policlinico San Matteo, Italy

## Abstract

Chikungunya virus (CHIKV) is an arthropod-borne virus of the family *Togaviridae* that is transmitted to humans by *Aedes* spp. mosquitoes. Its genome comprises a 12 kb single-strand positive-sense RNA. In the present study, we report the patterns of synonymous codon usage in 141 CHIKV genomes by calculating several codon usage indices and applying multivariate statistical methods. Relative synonymous codon usage (RSCU) analysis showed that the preferred synonymous codons were G/C and A-ended. A comparative analysis of RSCU between CHIKV and its hosts showed that codon usage patterns of CHIKV are a mixture of coincidence and antagonism. Similarity index analysis showed that the overall codon usage patterns of CHIKV have been strongly influenced by *Pan troglodytes* and *Aedes albopictus* during evolution. The overall codon usage bias was low in CHIKV genomes, as inferred from the analysis of effective number of codons (ENC) and codon adaptation index (CAI). Our data suggested that although mutation pressure dominates codon usage in CHIKV, patterns of codon usage in CHIKV are also under the influence of natural selection from its hosts and geography. To the best of our knowledge, this is first report describing codon usage analysis in CHIKV genomes. The findings from this study are expected to increase our understanding of factors involved in viral evolution, and fitness towards hosts and the environment.

## Introduction

Chikungunya virus (CHIKV), a member of the genus *alphavirus* of the family *Togaviridae*, is a small (60–70 nm), enveloped, single-strand positive-sense RNA virus. The genome is approximately 12 kb in size and comprises two open reading frames (ORFs) encoding non-structural and structural proteins, respectively [1]. The CHIKV genome is arranged in the order of 5-′cap-nsP1-nsP2-nsP3-nsP4-(junction region)-C-E3-E2-6K-E1-poly(A)-3′ [1]. Since the first isolation of CHIKV from a febrile individual in Tanzania in 1953 [2], CHIKV has caused several outbreaks in Asia, Africa, and Indian Ocean islands, emerging as a serious public health concern [3]–[6]. CHIKV infection is characterized by abrupt onset of high fever, headache, rashes, arthralgia and myalgia. The typical clinical sign of the disease is poly-arthralgia, which is a very painful condition affecting joints and may persist for several months to years in some cases [7]. Being an arthropod-borne virus, the mode of transmission is the mosquitoes of the *Aedes* spp. It is generally accepted that CHIKV originated from Africa, where it is primarily maintained in a yellow fever-like zoonotic sylvatic cycle and depends upon non-human primates and arboreal, peridomestic mosquitoes as reservoir hosts. However, the spread of CHIKV in Asia and urban endemics are associated with a dengue-like “human-mosquito-human” direct transmission cycle, where *A. aegypti* and *A. albopuctus* serve as primary transmission vectors and humans serve as hosts [7]–[9].

The genetic code comprises 64 codons that can be divided into 20 groups, where each group consists of one to six codons, and each group corresponds to each of the standard amino acids. Alternative codons within the same group coding for the same amino acid are often termed ‘synonymous’ codons, although their corresponding tRNAs might differ in their relative abundance in cells and in the speed by which they are recognized by the ribosome. This redundancy of the genetic code, in which most of the amino acids can be translated by more than one codon, represents a key step in modulating the efficiency and accuracy of protein production, while maintaining the same amino acid sequence of the protein. On the other hand, the synonymous codons are not chosen randomly both within and between genomes, which is referred to as codon usage bias [10],[11]. This phenomenon of synonymous codon usage bias has been studied in a wide range of organisms, from prokaryotes to eukaryotes and viruses [12]–[17]. Studies on codon usage have determined several factors that could influence codon usage patterns, including mutational pressure, natural or translational selection, secondary protein structure, replication and selective transcription, hydrophobicity and hydrophilicity of the protein and the external environment. Among these, the major factors responsible for codon usage variation among different organisms are considered to be compositional constraints under mutational pressure and natural selection [12], [18]–[20].

Previous studies on codon usage in different viruses have highlighted mutational pressure as the major factor in shaping codon usage patterns compared with natural selection [12], [21]–[23]; however, as our understanding of codon usage increases, it appears that although mutational pressure is still a major driving force, it is certainly not the only one when considering different types of RNA and DNA viruses [24]–[27]. Considering their comparatively small genome size and other viral features, such as dependence on host’s machinery for key process including replication, protein synthesis and transmission in comparison with prokaryotic and eukaryotic genomes, the interplay of codon usage among viruses and their hosts is expected to affect overall viral survival, fitness, evasion from host’s immune system and evolution [15], [28]. Therefore, knowledge of the codon usage in viruses can not only reveal information about molecular evolution, but also improve our understanding of the regulation of viral genes expression and aid vaccine design, where the efficient expression of viral proteins may be required to generate immunity. In the present study, we report the detailed codon usage data and analysis of various factors shaping the codon usage patterns in CHIKV genomes.

## Results and Discussion

### Nucleotide Composition Analysis of CHIKV Genomes

Codon usage bias, or preference for one type of codon over another, can be influenced greatly by the overall nucleotide composition of genomes [21]. Therefore, we first analyzed the nucleotide composition of coding sequences from CHIKV genomes. As shown in Table 1, the mean A% (28.91) was the highest, followed by similar composition of G% (25.75) and C% (25.19), with the U% being the lowest (20.16). The mean GC and AU compositions were 50.91% and 49.06% respectively. This appears to suggests there might be equal or almost equal distribution of A, U, G, and C nucleotides among codons of CHIKVs, with potentially more preference towards A-ended codons followed by G/C-ended codons. However, a clearer picture of overall nucleotide composition that could influence the codon usage preference in CHIKV genomes emerged from the analysis of the nucleotide composition of the third position of codons (A_3_, U_3_, G_3_, C_3_) and of GC_1_, GC_1,2_, GC_3_ and AU_3_ (Table 1). The mean C_3_ and G_3_ were the highest, followed by A_3_ and U_3_. The GC_3_ values ranged from 54.9% to 57.2%, with a mean of 55.86% and a standard deviation (SD) 0.40 compared with that of AU_3_, whose values ranged from 42.8% to 45.1%, with a mean of 44.14% and an SD of 0.41. The GC_1_ ranged from 50.6% to 53.8%, with a mean of 53.56% and an SD 0.27. The GC_1,2_ values ranged from 48.2% to 48.7%, with an average of 48.45% and an SD of 0.07. Therefore, from the initial nucleotide composition analysis, it is expected that G/C-ended codons might be preferred over A/U-ended codons in CHIKV genomes.

**Table 1:** Nucleotide composition analysis of CHIKV genomes (%).

No	A	U	G	C	A_3_	U_3_	G_3_	C_3_	AU	GC	GC_1_	GC_2_	AU_3_	GC_3_	GC_12_	ENC
1	29.0	19.9	25.7	25.4	27.1	16.7	26.8	29.3	48.9	51.1	53.7	43.5	43.8	56.2	48.6	55.13
2	28.9	20.0	25.7	25.4	27.0	16.7	26.9	29.3	48.9	51.1	53.7	43.3	43.7	56.3	48.5	55.11
3	28.9	20.3	25.9	24.9	26.7	17.7	27.4	28.2	49.2	50.8	53.5	43.3	44.4	55.6	48.4	55.66
4	28.9	19.9	25.8	25.4	26.7	16.9	27.2	29.2	48.8	51.2	53.8	43.4	43.6	56.4	48.6	55.09
5	28.9	20.1	25.7	25.3	27.0	17.2	27.0	28.9	49.0	51.0	53.7	43.4	44.2	55.9	48.6	55.54
6	29.0	20.1	25.7	25.3	27.2	16.9	26.7	29.2	49.1	51.0	53.7	43.4	44.1	55.9	48.6	55.33
7	28.8	20.4	25.9	24.9	26.6	17.9	27.4	28.1	49.2	50.8	53.5	43.3	44.5	55.5	48.4	55.95
8	28.8	20.4	25.9	24.9	26.6	17.9	27.4	28.1	49.2	50.8	53.5	43.3	44.5	55.5	48.4	55.94
9	28.8	20.4	25.9	24.9	26.6	17.9	27.3	28.1	49.2	50.8	53.5	43.4	44.5	55.5	48.5	55.91
10	28.7	20.1	25.9	25.2	26.0	17.2	27.8	28.9	48.8	51.2	53.4	43.4	43.2	56.7	48.4	54.93
11	28.7	20.1	25.9	25.3	26.2	17.0	27.7	29.1	48.8	51.2	53.4	43.4	43.2	56.8	48.4	54.81
12	28.7	20.2	25.9	25.2	26.2	17.3	27.7	28.8	48.9	51.1	53.4	43.4	43.5	56.5	48.4	54.97
13	28.9	20.4	25.8	24.9	26.8	18.0	27.3	28.0	49.3	50.7	53.5	43.3	44.8	55.2	48.4	55.88
14	28.9	20.4	25.8	24.9	26.8	18.0	27.2	28.0	49.3	50.7	53.5	43.3	44.8	55.2	48.4	55.84
15	28.8	20.5	25.9	24.8	26.6	18.2	27.5	27.7	49.3	50.6	53.5	43.2	44.8	55.3	48.4	56.09
16	29.0	20.0	25.7	25.4	27.1	16.9	26.8	29.1	49.0	51.0	53.7	43.4	44.0	55.9	48.6	55.24
17	28.8	20.5	25.9	24.8	26.6	18.1	27.6	27.7	49.3	50.7	53.5	43.3	44.7	55.3	48.4	56.04
18	29.0	20.2	25.7	25.1	27.0	17.5	26.9	28.6	49.2	50.8	53.6	43.4	44.5	55.5	48.5	55.52
19	28.7	20.1	25.9	25.3	26.2	17.1	27.7	29.1	48.8	51.2	53.4	43.4	43.3	56.7	48.4	54.92
20	28.7	20.0	26.0	25.3	26.1	17.0	27.8	29.2	48.7	51.3	53.4	43.5	43.1	56.9	48.5	54.66
21	28.9	20.2	25.7	25.2	26.2	17.1	27.6	29.1	49.1	51.2	53.4	43.5	43.3	56.7	48.5	54.85
22	29.0	20.1	25.7	25.3	27.0	17.3	27.0	28.8	49.1	50.9	53.6	43.5	44.3	55.8	48.6	55.53
23	28.8	20.5	25.9	24.8	26.7	18.1	27.5	27.8	49.3	50.7	53.4	43.3	44.8	55.3	48.4	56.08
24	28.7	20.1	25.9	25.4	26.0	17.1	27.7	29.2	48.8	51.3	53.5	43.4	43.1	56.9	48.5	54.80
25	29.1	20.0	25.6	25.3	27.2	17.2	26.7	28.9	49.1	50.9	53.6	43.5	44.4	55.6	48.6	55.48
26	28.8	20.5	25.9	24.8	26.6	18.2	27.4	27.7	49.3	50.6	53.5	43.3	44.8	55.1	48.4	56.11
27	28.8	20.5	25.9	24.8	26.6	18.1	27.5	27.8	49.3	50.7	53.5	43.2	44.7	55.3	48.4	56.02
28	28.9	20.5	25.9	24.8	26.7	18.1	27.5	27.8	49.4	50.7	53.5	43.2	44.8	55.3	48.4	56.02
29	29.1	20.0	25.6	25.3	27.4	16.9	26.7	29.1	49.1	50.9	53.7	43.4	44.3	55.8	48.6	55.08
30	28.9	20.0	25.7	25.3	27.0	16.9	26.9	29.2	48.9	51.0	53.5	43.4	43.9	56.2	48.5	55.32
31	28.8	20.5	25.9	24.8	26.5	18.2	27.6	27.7	49.3	50.7	53.4	43.4	44.7	55.3	48.4	56.15
32	28.7	20.0	26.0	25.4	25.9	16.9	27.9	29.3	48.7	51.3	53.4	43.4	42.8	57.2	48.4	54.57
33	28.6	20.0	26.0	25.3	26.0	17.0	27.8	29.2	48.6	51.3	53.6	43.4	43.0	57.0	48.5	54.56
34	28.8	20.6	25.9	24.7	26.6	18.4	27.5	27.5	49.4	50.6	53.5	43.3	45.0	55.0	48.4	56.26
35	28.8	20.6	25.9	24.7	26.6	18.3	27.6	27.5	49.4	50.6	53.4	43.3	44.9	55.1	48.4	56.22
36	28.8	20.6	25.9	24.7	26.6	18.3	27.5	27.6	49.4	50.6	53.4	43.3	44.9	55.1	48.4	56.28
37	29.0	20.1	25.7	25.3	26.9	17.3	27.0	28.8	49.1	50.9	53.6	43.4	44.2	55.7	48.5	55.55
38	28.7	20.1	25.9	25.2	26.1	17.2	27.7	29.0	48.8	51.1	53.4	43.3	43.3	56.7	48.4	54.55
39	29.0	20.0	25.7	25.3	26.9	17.1	27.0	29.1	49.0	51.0	53.6	43.3	44.0	56.0	48.5	55.45
40	29.0	20.0	25.7	25.3	26.9	17.1	27.0	29.1	49.0	51.0	53.6	43.3	44.0	56.0	48.5	55.45
41	28.9	20.1	25.7	25.3	26.4	16.8	27.1	29.7	49.0	51.4	53.8	43.7	43.2	56.8	48.8	55.44
42	28.8	19.7	25.9	25.6	26.7	18.4	27.4	27.5	48.5	50.5	53.4	43.3	45.1	54.9	48.4	56.25
43	28.8	20.7	25.9	24.6	26.9	17.2	26.9	29.0	49.5	50.9	53.6	43.3	44.1	55.9	48.5	55.51
44	29.0	20.1	25.7	25.3	26.7	18.4	27.3	27.5	49.1	50.5	53.4	43.3	45.1	54.9	48.4	56.28
45	28.8	20.7	25.9	24.6	26.7	18.4	27.4	27.5	49.5	50.6	53.5	43.3	45.1	54.9	48.4	56.23
46	28.8	20.7	25.9	24.6	26.8	17.2	27.0	29.0	49.5	51.0	53.6	43.3	44.0	56.0	48.5	55.49
47	29.0	20.1	25.7	25.3	26.9	17.2	26.9	28.9	49.1	50.9	53.6	43.3	44.1	55.9	48.5	55.53
48	29.0	20.1	25.7	25.3	27.0	17.1	26.9	29.0	49.1	51.0	53.7	43.3	44.1	55.9	48.5	55.42
49	29.0	20.0	25.7	25.3	26.8	17.2	27.0	29.0	49.0	50.9	53.6	43.3	44.0	55.9	48.5	55.46
50	29.0	20.0	25.7	25.3	26.9	17.1	27.0	29.1	49.0	51.0	53.6	43.3	44.0	56.0	48.5	55.46
51	28.9	20.1	25.7	25.3	26.8	17.2	27.0	29.0	49.0	51.0	53.6	43.3	44.0	56.0	48.5	55.46
52	29.0	20.1	25.7	25.3	26.8	17.2	27.0	29.0	49.1	50.9	53.6	43.3	44.0	55.9	48.5	55.51
53	29.0	20.1	25.7	25.3	26.9	17.1	27.0	29.0	49.1	51.0	53.6	43.4	44.0	56.0	48.5	55.44
54	29.0	20.1	25.7	25.3	26.8	17.2	27.0	29.0	49.1	51.0	53.6	43.3	44.0	56.0	48.5	55.47
55	28.9	20.1	25.7	25.3	26.9	17.2	26.9	29.0	49.0	50.9	53.5	43.3	44.1	55.9	48.4	55.46
56	29.0	20.1	25.7	25.3	26.8	17.2	27.0	28.9	49.1	51.0	53.6	43.4	44.0	55.9	48.5	55.53
57	28.9	20.1	25.7	25.3	26.9	17.2	27.0	28.9	49.0	50.9	53.6	43.3	44.1	55.9	48.5	55.52
58	29.0	20.1	25.7	25.3	26.9	17.2	26.9	29.0	49.1	51.0	53.6	43.3	44.1	55.9	48.5	55.43
59	29.0	20.1	25.7	25.3	26.9	17.3	27.0	28.9	49.1	50.9	53.6	43.3	44.2	55.9	48.5	55.53
60	29.0	20.1	25.7	25.3	26.9	17.3	27.0	28.9	49.1	50.9	53.7	43.2	44.2	55.8	48.5	55.54
61	29.0	20.1	25.7	25.3	26.9	17.3	26.9	28.9	49.1	50.9	53.6	43.2	44.2	55.8	48.4	55.49
62	29.0	20.1	25.7	25.3	26.8	17.1	27.0	29.0	49.1	51.0	53.6	43.3	43.9	56.0	48.5	55.44
63	29.0	20.0	25.7	25.3	26.9	17.0	27.0	29.1	49.0	51.0	53.6	43.3	43.9	56.1	48.5	55.45
64	29.0	20.1	25.7	25.3	26.8	17.2	27.0	29.0	49.1	50.9	53.6	43.3	44.0	56.0	48.5	55.50
65	29.0	20.1	25.7	25.3	26.9	17.2	26.9	29.0	49.1	51.0	53.6	43.3	44.1	55.9	48.5	55.50
66	29.0	20.1	25.7	25.3	26.9	17.2	27.0	29.0	49.1	50.9	53.6	43.3	44.1	55.9	48.5	55.49
67	28.7	20.1	25.9	25.3	26.9	17.1	26.9	29.1	48.8	51.0	53.6	43.4	44.0	56.0	48.5	55.49
68	29.0	20.1	25.7	25.3	26.8	17.2	27.0	29.0	49.1	51.0	53.6	43.3	44.0	56.0	48.5	55.50
69	29.0	20.1	25.7	25.3	26.9	17.2	26.9	29.0	49.1	50.9	53.5	43.3	44.1	55.9	48.4	55.52
70	29.1	20.0	25.9	25.0	27.1	16.9	27.1	28.9	49.1	50.9	53.7	43.2	44.0	56.0	48.5	55.11
71	28.7	20.6	26.0	24.7	26.9	17.2	27.0	28.9	49.3	50.9	53.6	43.3	44.1	55.9	48.5	55.42
72	29.0	20.1	25.6	25.2	26.6	18.3	27.5	27.7	49.1	50.6	53.4	43.4	44.9	55.1	48.4	56.28
73	29.0	20.1	25.7	25.3	26.9	17.2	27.0	28.9	49.1	50.9	53.6	43.3	44.1	55.9	48.5	55.57
74	29.0	20.1	25.7	25.2	26.9	17.2	27.0	28.9	49.1	51.0	53.6	43.3	44.1	55.9	48.5	55.55
75	29.0	20.1	25.7	25.3	26.9	17.3	27.0	28.9	49.1	50.9	53.6	43.3	44.2	55.8	48.5	55.55
76	29.0	20.1	25.7	25.3	26.9	17.2	26.9	28.9	49.1	50.9	53.6	43.2	44.1	55.9	48.4	55.49
77	29.0	20.1	25.7	25.2	26.8	17.3	27.0	28.9	49.1	51.0	53.7	43.4	44.1	55.9	48.6	55.61
78	28.9	20.1	25.7	25.3	26.9	17.2	27.0	29.0	49.0	51.0	53.7	43.4	44.1	56.0	48.6	55.58
79	29.0	20.1	25.7	25.2	26.9	17.3	27.0	28.9	49.1	50.9	53.6	43.3	44.2	55.9	48.5	55.58
80	28.9	20.1	25.7	25.2	26.9	17.3	27.0	28.9	49.0	50.9	53.5	43.4	44.2	55.9	48.5	55.55
81	28.9	20.1	25.7	25.3	26.8	17.2	27.0	29.0	49.0	51.0	53.6	43.3	44.0	56.0	48.5	55.55
82	28.9	20.1	25.7	25.3	26.8	17.2	27.0	29.0	49.0	50.9	53.6	43.3	44.0	56.0	48.5	55.51
83	29.0	20.1	25.6	25.2	27.0	17.3	26.8	28.9	49.1	50.8	53.5	43.3	44.3	55.7	48.4	55.63
84	29.0	20.1	25.7	25.2	26.8	17.3	27.0	28.8	49.1	50.9	53.6	43.3	44.1	55.9	48.5	55.60
85	28.9	20.1	25.7	25.3	26.9	17.3	27.0	28.9	49.0	50.9	53.6	43.3	44.2	55.8	48.5	55.48
86	28.9	20.1	25.7	25.3	26.8	17.3	27.0	28.8	49.0	50.9	53.6	43.3	44.1	55.8	48.5	55.61
87	29.0	20.1	25.7	25.3	26.8	17.2	27.0	29.0	49.1	50.9	53.6	43.3	44.0	56.0	48.5	55.51
88	28.9	20.1	25.7	25.2	27.0	17.2	26.8	29.0	49.0	50.9	53.6	43.3	44.2	55.8	48.5	55.53
89	29.0	20.1	25.6	25.3	26.8	17.3	27.0	28.9	49.1	50.9	53.6	43.3	44.1	56.0	48.5	55.58
90	29.0	20.1	25.6	25.3	26.9	17.2	27.0	29.0	49.1	50.9	53.6	43.2	44.1	55.9	48.4	55.38
91	29.0	20.1	25.6	25.3	27.0	17.3	26.8	28.9	49.1	50.9	53.6	43.3	44.3	55.8	48.5	55.54
92	29.0	20.1	25.7	25.3	27.0	17.2	26.9	29.0	49.1	50.9	53.6	43.3	44.2	55.8	48.5	55.53
93	29.0	20.1	25.7	25.3	26.9	17.2	27.0	29.0	49.1	50.9	53.6	43.2	44.1	56.0	48.4	55.44
94	29.0	20.1	25.7	25.3	26.9	17.2	27.0	28.9	49.1	50.9	53.6	43.2	44.1	55.9	48.4	55.42
95	28.9	20.1	25.7	25.2	26.8	17.3	27.0	28.9	49.0	50.9	53.6	43.3	44.1	55.9	48.5	55.59
96	29.0	20.1	25.7	25.2	26.9	17.2	27.0	29.0	49.1	50.9	53.6	43.3	44.1	55.9	48.5	55.43
97	29.0	20.1	25.7	25.3	26.8	17.3	27.0	28.9	49.1	50.9	53.5	43.3	44.1	55.9	48.4	55.57
98	28.9	20.2	25.7	25.2	26.9	17.2	27.0	29.0	49.1	50.9	53.6	43.2	44.1	56.0	48.4	55.42
99	29.0	20.1	25.7	25.3	26.8	17.3	27.0	28.9	49.1	50.9	53.6	43.3	44.1	55.9	48.5	55.59
100	28.9	20.2	25.7	25.2	27.0	17.2	26.9	29.0	49.1	50.9	53.6	43.3	44.2	55.9	48.5	55.41
101	29.0	20.1	25.7	25.3	26.7	17.3	27.1	28.9	49.1	50.9	53.6	43.3	44.0	56.0	48.5	55.56
102	29.0	20.1	25.7	25.3	26.8	17.3	27.0	28.9	49.1	50.9	53.5	43.2	44.1	56.0	48.4	55.59
103	29.0	20.1	25.7	25.3	26.8	17.3	27.0	28.9	49.1	50.9	53.6	43.3	44.1	55.9	48.5	55.63
104	28.9	20.1	25.7	25.2	26.8	17.3	27.0	28.9	49.0	51.0	53.6	43.3	44.1	55.9	48.5	55.58
105	28.9	20.1	25.7	25.3	26.8	17.2	27.0	29.0	49.0	51.0	53.7	43.4	44.0	56.0	48.6	55.56
106	28.9	20.1	25.7	25.2	26.8	17.3	27.0	28.8	49.0	50.9	53.6	43.3	44.1	55.8	48.5	55.60
107	28.9	20.1	25.7	25.2	26.8	17.3	27.0	28.9	49.0	50.9	53.6	43.3	44.1	55.9	48.5	55.63
108	28.9	20.1	25.7	25.3	26.8	17.3	27.0	28.9	49.0	50.9	53.6	43.3	44.1	55.9	48.5	55.65
109	28.9	20.1	25.7	25.2	26.8	17.3	27.0	28.9	49.0	50.9	53.6	43.3	44.1	55.9	48.5	55.65
110	28.9	20.1	25.7	25.2	26.8	17.3	27.0	28.9	49.0	50.9	53.6	43.3	44.1	55.9	48.5	55.63
111	28.9	20.1	25.7	25.2	26.8	17.3	27.0	28.9	49.0	50.9	53.6	43.3	44.1	55.9	48.5	55.66
112	28.9	20.1	25.7	25.2	26.8	17.3	27.0	28.8	49.0	50.9	53.6	43.3	44.1	55.8	48.5	55.67
113	28.9	20.1	25.7	25.2	26.8	17.3	27.0	28.9	49.0	50.9	53.6	43.3	44.1	55.8	48.5	55.63
114	28.9	20.1	25.7	25.2	26.8	17.2	27.0	28.9	49.0	50.9	53.6	43.3	44.0	56.0	48.5	55.58
115	29.0	20.2	25.6	25.2	26.9	17.4	26.9	28.8	49.2	50.8	53.5	43.3	44.3	55.7	48.4	55.62
116	29.0	20.1	25.6	25.2	27.0	17.3	26.8	28.9	49.1	50.9	53.6	43.3	44.3	55.7	48.5	55.59
117	28.9	20.1	25.7	25.2	26.3	17.0	27.2	29.5	49.0	51.4	53.8	43.7	43.3	56.7	48.8	55.49
118	28.8	19.8	25.9	25.5	26.8	17.4	27.0	28.8	48.6	50.9	53.6	43.3	44.2	55.8	48.5	55.63
119	28.9	20.1	25.7	25.3	26.8	17.3	27.0	28.9	49.0	50.9	53.6	43.3	44.1	55.9	48.5	55.59
120	28.9	20.1	25.7	25.2	26.8	17.3	27.0	28.8	49.0	50.9	53.6	43.4	44.1	55.8	48.5	55.63
121	28.9	20.2	25.7	25.2	26.8	17.4	27.0	28.8	49.1	50.9	53.5	43.3	44.2	55.8	48.4	55.66
122	28.9	20.2	25.7	25.2	26.8	17.5	27.0	28.7	49.1	50.9	53.6	43.3	44.3	55.7	48.5	55.79
123	28.9	20.1	25.7	25.2	26.8	17.4	27.1	28.8	49.0	50.9	53.6	43.4	44.2	55.9	48.5	55.55
124	28.9	20.2	25.7	25.2	26.7	17.5	27.1	28.8	49.1	50.9	53.6	43.3	44.2	55.9	48.5	55.84
125	28.9	20.1	25.7	25.2	26.8	17.3	27.1	28.8	49.0	51.0	53.6	43.3	44.1	56.0	48.5	55.55
126	28.9	20.1	25.7	25.2	26.8	17.4	27.0	28.8	49.0	50.9	53.6	43.3	44.2	55.8	48.5	55.77
127	28.9	20.1	25.7	25.2	26.8	17.3	27.0	28.9	49.0	50.9	53.7	43.3	44.1	55.9	48.5	55.68
128	28.9	20.2	25.7	25.2	26.8	17.4	27.0	28.8	49.1	50.9	53.6	43.4	44.2	55.8	48.5	55.54
129	29.0	20.1	25.7	25.3	27.1	16.9	26.9	29.1	49.1	50.9	53.4	43.3	44.0	56.0	48.4	55.28
130	28.7	20.6	26	24.7	26.5	18.3	27.6	27.6	49.3	50.7	53.5	43.3	44.8	55.2	48.4	56.19
131	28.9	20.1	25.7	25.2	26.9	17.4	27.0	28.8	49.0	50.9	53.7	43.3	44.3	55.7	48.5	55.60
132	28.9	20.1	25.7	25.2	26.9	17.3	27.0	28.8	49.0	50.9	53.7	43.3	44.2	55.8	48.5	55.60
133	28.9	20.1	25.7	25.2	26.9	17.3	27.0	28.8	49.0	50.9	53.7	43.3	44.2	55.8	48.5	55.60
134	28.9	20.1	25.7	25.3	26.9	17.3	27.0	28.8	49.0	50.9	53.7	43.3	44.2	55.8	48.5	55.59
135	28.9	20.1	25.7	25.3	26.9	17.3	27.0	28.8	49.0	50.9	53.7	43.3	44.2	55.8	48.5	55.59
136	28.9	20.1	25.7	25.2	26.9	17.3	27.0	28.8	49.0	50.9	53.7	43.3	44.2	55.8	48.5	55.61
137	28.9	20.1	25.7	25.2	26.9	17.3	27.0	28.8	49.0	50.9	53.7	43.3	44.2	55.8	48.5	55.61
138	28.8	20.6	26.0	24.7	26.6	18.2	27.5	27.7	49.4	50.6	53.3	43.2	44.8	55.3	48.3	56.41
139	28.8	20.6	26.0	24.7	26.5	18.2	27.6	27.7	49.4	50.6	50.6	50.6	44.7	55.3	48.3	56.41
140	29.0	20.1	25.9	25.3	27.0	17.1	26.8	29.1	49.1	51.0	53.6	43.4	44.1	55.9	48.5	55.39
141	28.9	20.0	25.7	25.3	27.3	17.0	26.7	29.0	48.9	50.9	53.7	43.3	44.3	55.7	48.5	55.25
Mean	28.91	20.16	25.75	25.19	26.78	17.36	27.10	28.76	49.07	50.91	53.56	43.38	44.14	55.86	48.45	55.56
SD	0.10	0.18	0.10	0.20	0.25	0.39	0.27	0.47	0.16	0.16	0.27	0.62	0.41	0.40	0.07	0.34

SD: Standard deviation.

### Relative Synonymous Codon Usage (RSCU) Analysis of CHIKV

**Figure 1:**
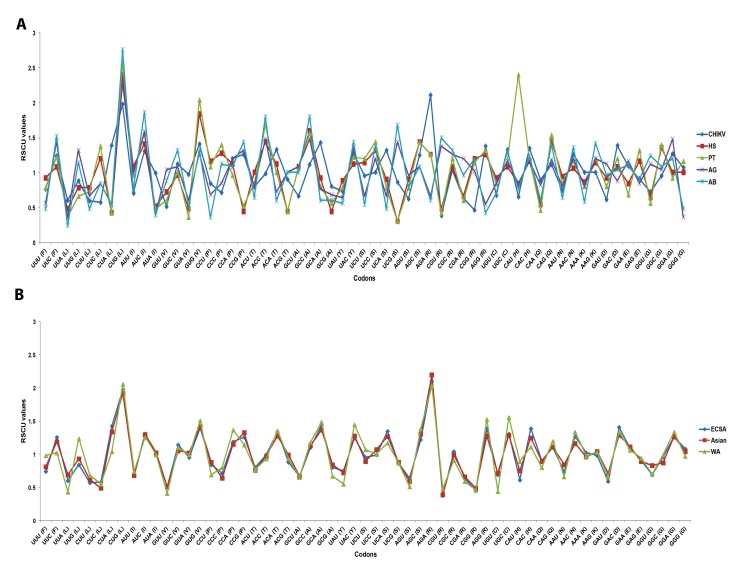
Comparative analysis of relative synonymous codon usage (RSCU) patterns. (**A**) between chikungunya virus (CHIKV), *Homo sapiens* (HS), *Pan troglodytes* (PT) and *Aedes aegypti* (AG) and *Aedes albopictus* (AB). (**B**) between east central south African (ECSA), Asian and West African (WA) genotypes of CHIKV.

To determine the patterns of synonymous codon usage and to what extent G/C-ended codons might be preferred, we performed RSCU analysis and calculated the RSCU values. Among the 18 most abundantly used codons in CHIKV genomes, eleven (UUC, CUG, AUC, GUG, CCG, UAC, UGC, CAC, CAG, AAC and GAC) were G/C-ended (C-ended: 7; G-ended: 4) and the remaining seven (ACA, GCA, UCA, AGA, AAA, GAA, GGA) were A-ended codons; none of the preferred codons were U-ended (Figure 1A and Table 2). From RSCU analysis, we observed that CHIKV exhibits comparatively higher codon usage bias towards G/C- and less towards A-ended codons. However, it is also interesting to note that the mean GC% and AU% values are very similar (Table 1), yet the G/C- ending codons were used in a comparatively biased manner, indicating that the G/C content at the third position of the codons influenced the shaping of the overall synonymous codons usage patterns. The overall general trend of the 59 synonymous codon usages was also relatively consistent among different genotypes of CHIKV, indicating that the evolutionary processes of the three genotypes of CHIKV are restricted by the synonymous codon usage pattern to some extent (Figure 1B and Table 2). Furthermore, analysis of over- and under-represented codons showed that codons with an RSCU>1.6 are infrequently observed in CHIKV genomes. The RSCU values of the majority of preferred and non-preferred codons fell between 0.6 and 1.6. We further divided the RSCU data into three groups; (A) codons with RSCU<0.6 (under-represented), (B) codons with RSCU values between 0.6 and 1.6 (unbiased/randomly represented), and (C) codons with RSCU values >1.6 (over-represented). Among 59 codons, only CUG (Leu) and AGA (Arg) had an RSCU>1.6. However, the under-represented codons (RSCU<0.6), were identified as follows: CUU, CUC for Leu, GUU for Val, and CGU, CGG for Arg. The remaining 52 codons had RSCU values between 0.6–1.6 (Figure 1 and Table 2). These findings suggested that despite being an RNA virus with a high mutation rate in its lifecycle, CHIKV has evolved to form a relatively stable genetic composition at some specific levels of synonymous codon usage. This was further confirmed by ENC and CAI analysis as discussed in coming sections. Combining nucleotide composition and RSCU analysis, we deduced that the selection for preferred codons has been mostly influenced by compositional constraints, which also accounts for the presence of mutational pressure. However, we suspect that the compositional constraints may not be the sole factor associated with codon usage patterns in CHIKV, because although the overall RSCU values could reveal the codon usage pattern for the genomes, it may hide the codon usage variation among different genes in a genome [29].

**Table 2:** The synonymous codon usage patterns of CHIKV, its hosts and transmission vectors.

		RSCU										RSCU							
AA	Codon	CHIKV				Hosts & Vectors				AA	Codon	CHIKV				Hosts & Vectors			
		Overall	ECSA	Asian	WA	*HS*	*PT*	*AG*	*AB*			Overall	ECSA	Asian	WA	*HS*	*PT*	*AG*	*AB*
Phe^b^^,^ c	UUU	0.76	0.74	0.81	0.98	0.92	0.78	0.56	0.48	Ser^a^^,^ c	UCU	0.95	0.95	0.89	1.07	1.14	1.20	0.66	0.54
	**UUC**	**1.24**	**1.26**	**1.19**	**1.02**	**1.08**	**1.22**	**1.44**	**1.52**		UCC	1.00	0.99	1.07	1.00	1.32	1.44	1.20	1.38
Leu^b^^,^ d	UUA	0.60	0.60	0.69	0.43	0.48	0.36	0.36	0.24		**UCA**	**1.32**	1.35	1.27	1.17	0.90	0.78	0.66	0.48
	UUG	0.88	0.84	0.93	1.24	0.78	0.66	1.32	1.14		UCG	0.86	0.86	0.88	0.87	0.30	0.30	**1.44**	**1.68**
	CUU	0.59	0.57	0.62	0.68	0.78	0.72	0.66	0.48		AGU	0.62	0.64	0.59	0.51	0.90	0.78	0.96	0.78
	CUC	0.57	0.58	0.49	0.55	1.20	1.38	0.84	0.84		AGC	1.25	1.22	**1.31**	**1.39**	**1.44**	**1.44**	1.08	1.08
	CUA	1.39	1.43	1.34	1.04	0.42	0.42	0.54	0.54	Arg^a^^,^ c	**AGA**	**2.11**	**2.10**	**2.20**	**2.04**	**1.26**	1.26	0.66	0.60
	**CUG**	**1.98**	**1.98**	**1.92**	**2.06**	**2.40**	**2.58**	**2.28**	**2.76**		CGU	0.38	0.38	0.39	0.48	0.48	0.42	**1.38**	**1.50**
Ile^b^^,^ c	AUU	0.70	0.70	0.68	0.74	1.08	0.96	0.99	0.75		CGC	1.03	1.04	0.99	0.91	1.08	1.20	1.26	1.32
	**AUC**	**1.31**	**1.31**	**1.30**	**1.26**	**1.41**	**1.56**	**1.59**	**1.86**		CGA	0.63	0.63	0.66	0.59	0.66	0.60	1.20	0.96
	AUA	0.99	0.99	1.02	0.99	0.51	0.48	0.39	0.39		CGG	0.46	0.45	0.49	0.46	1.20	1.14	1.02	1.20
Val^b^^,^ c	GUU	0.51	0.51	0.51	0.41	0.72	0.60	1.04	0.88		AGG	1.38	1.39	1.28	1.53	1.26	**1.32**	0.54	0.42
	GUC	1.12	1.14	1.05	1.10	0.96	1.00	1.08	1.32	Cys^b^^,^ c	UGU	0.67	0.69	0.71	0.44	0.92	0.84	0.84	0.70
	GUA	0.97	0.95	1.02	0.99	0.48	0.36	0.60	0.52		**UGC**	**1.33**	**1.31**	**1.29**	**1.56**	**1.08**	**1.16**	**1.16**	**1.30**
	**GUG**	**1.41**	**1.40**	**1.42**	**1.51**	**1.84**	**2.04**	**1.28**	**1.32**	His^b^^,^ c	CAU	0.65	0.61	0.75	0.89	0.84	**2.40**	0.84	0.76
Pro^b^^,^ d	CCU	0.84	0.84	0.88	0.69	1.16	1.08	0.68	0.36		**CAC**	**1.35**	**1.39**	**1.25**	**1.11**	**1.16**	1.20	**1.16**	**1.24**
	CCC	0.71	0.71	0.64	0.80	**1.28**	**1.40**	0.84	1.12	Gln^b^^,^ c	CAA	0.89	0.90	0.89	0.80	0.54	0.46	0.82	0.60
	CCA	1.20	1.19	1.15	**1.37**	1.12	0.96	1.20	1.08		**CAG**	**1.11**	**1.10**	**1.11**	**1.20**	**1.46**	**1.54**	**1.18**	**1.40**
	**CCG**	**1.26**	**1.26**	**1.33**	1.14	0.44	0.52	**1.32**	**1.44**	Asn^b^^,^ c	AAU	0.74	0.73	0.84	0.66	0.94	0.84	0.80	0.64
Thr^a^^,^ c	ACU	0.79	0.80	0.76	0.77	1.00	0.84	0.80	0.64		**AAC**	**1.26**	**1.27**	**1.16**	**1.34**	**1.06**	**1.16**	**1.20**	**1.36**
	ACC	0.98	0.99	0.97	0.93	**1.44**	**1.68**	**1.48**	**1.80**	Lys^a^^,^ d	AAA	1.00	**1.02**	0.96	0.97	0.86	0.80	0.80	0.58
	**ACA**	**1.33**	**1.34**	**1.28**	**1.36**	1.12	1.00	0.72	0.60		**AAG**	**1.00**	0.98	**1.04**	**1.03**	**1.14**	**1.20**	**1.20**	**1.42**
	ACG	0.90	0.88	0.99	0.93	0.44	0.44	1.00	1.00	Asp^b^^,^ c	GAU	0.61	0.59	0.71	0.66	0.92	0.80	**1.12**	0.96
Ala^a^^,^ c	GCU	0.66	0.66	0.67	0.65	1.08	1.08	1.08	1.00		**GAC**	**1.39**	**1.41**	**1.29**	**1.34**	**1.08**	**1.20**	0.88	**1.04**
	GCC	1.11	1.10	1.13	1.18	**1.60**	**1.56**	**1.48**	**1.80**	Glu^a^^,^ d	**GAA**	**1.09**	1.09	1.11	1.06	0.84	0.68	**1.16**	**1.10**
	**GCA**	**1.43**	**1.44**	**1.37**	**1.49**	0.92	0.80	0.76	0.60		GAG	0.91	0.91	0.89	0.94	**1.16**	**1.32**	0.84	0.90
	GCG	0.80	0.80	0.84	0.67	0.44	0.56	0.68	0.60	Gly^a^^,^ c	GGU	0.72	0.69	0.83	0.70	0.64	0.56	1.12	**1.24**
Tyr^b^^,^ c	UAU	0.73	0.75	0.72	0.55	0.88	0.78	0.64	0.56		GGC	0.95	0.96	0.87	0.99	**1.36**	**1.40**	1.04	1.08
	**UAC**	**1.27**	**1.25**	**1.28**	**1.45**	**1.12**	**1.22**	**1.36**	**1.44**		**GGA**	**1.27**	**1.26**	**1.27**	**1.34**	1.00	0.92	**1.48**	1.20
											GGG	1.07	1.08	1.04	0.97	1.00	1.16	0.36	0.48

AA: amino acid, HS: *H. sapiens*, AG: *A. aegypti*, AB: *A. albopictus*, PT: *P. troglodytes*. Preferred codons of CHIKV, *H. sapiens*, *A. aegypti*, *A. albopictus and P. troglodytes* are shown in bold.

a Amino acids with A/U-ended preferred codons in CHIKV.

b Amino acids with G/C-ended preferred codons in CHIKV.

c Amino acids with A/U-ended preferred codons in CHIKV.

d Amino acids with G/C-ended preferred codons in CHIKV.

### Codon Usage Bias among CHIKV

To quantify the extent of variation in codon usage among different genomes of CHIKV arising from different geographical regions and genotypes, the ENC values for each genome were calculated. The ENC values among CHIKV genomes ranged from 54.55 to 56.41, with a mean of 55.56 and an SD of 0.34 (Table 1). An average value of 55.56 (ENC>40) represents stable ENC values and indicates a relatively conserved genomic composition among different CHIKV genomes. In general, there is an inverse relationship between ENC and gene expression; i.e., a lower ENC value indicates a higher codon usage preference and higher gene expression and vice versa [30]. Our results show that the overall codon usage bias and gene expression among different CHIKV genomes is lower, slightly biased and would be mainly affected by the base composition. Previous studies on codon usage analysis among other RNA viruses, such as bovine viral diarrhea virus (ENC: 50.91) [22], classical swine fever virus (ENC = 51.7) [17] and HCV (ENC = 52.62) [31], have also reported lower codon usage bias. The same is also true in the case of arthropod-borne RNA viruses, including West Nile virus (ENC: 53.81) [15] and dengue virus (DENV) (ENC: 49.70: DENV-1; 48.78: DENV-2; 49.52: DENV-3; and 50.81: DENV-4) [14]. A possible explanation for the weak codon bias of RNA viruses is that it might be advantageous for efficient replication in host cells, with potentially distinct codon preferences [21].

The codon adaptation index (CAI) is often used as measure of level of gene expression and to assess the adaptation of viral genes to their hosts. Highly expressed genes exhibit a strong bias for particular codons in many bacteria and small eukaryotes. In comparison to the ENC, which is another way of calculating codon usage bias and measures deviation from a uniform bias (null hypothesis), CAI measures the deviation of a given protein coding gene sequence with respect to a reference set of genes [32]. Here, we calculated the CAI values of coding sequences from CHIKV genomes. The CAI values ranged from 0.21 to 0.22, with a mean value of 0.22 and an SD of 0.001 (data not shown). The mean CAI value was low, indicating low codon usage bias and expression levels, which agreed with the ENC analysis.

### Relationship between Codon Usage Patterns of CHIKV and its Hosts

Being parasitic organisms, it can be expected that the codon usage patterns of viruses would be affected by its hosts to some extent [33]. For instance, the codon usage pattern of poliovirus is reported to be mostly coincident with that of its host [34], while the codon usage pattern of hepatitis A was reported to be antagonistic to that of its host [35]. We therefore computed and compared the codon usage of CHIKV with its two hosts (*Homo sapiens* and *Pan troglodytes*), and transmission vectors (*A. aegypti* and *A. albopictus*). The results showed that the codon usage patterns of CHIKV were a mixture of coincidence and antagonism to its hosts and vectors (Table 2). In detail, the preferred codons for 12 out of 18 amino acids were common between CHIKV and *H. sapiens*. This included UUC (Phe), CUG (Leu), AUC (Ile), GUG (Val), UAC (Tyr), AGA (Arg), UGC (Cys), CAC (His), CAG (Gln), AAC (Asn), AAG (Lys) and GAC (Asp). Furthermore, all common preferred codons between CHIKV and *H. sapiens* were G/C- ended (C-ended: 7; G-ended: 4), with exception of an A-ended preferred codon for amino acid Arg. Similarly, preferred codons for 10 out of 18 amino acids were common between CHIKV and *P. troglodytes*. In case of the two transmission vectors, 10 out of 18 preferred codons were common among both mosquito species and CHIKV. It is also interesting to note that, except for amino acid Arg, the remaining 10 highly preferred codons were same among CHIKV, *H. sapiens*, *A. aegypti* and *A. albopictus*. Moreover, the preferred codon usage profiles of *A. aegypti* and *A. albopictus* were also very similar: 16 out of 18 preferred codons were common between, with exceptions for the preferred codons for Asp and Gly (Table 2). These results indicated that selection pressures from hosts and vectors have influenced the codon usage pattern of CHIKV and the possible fitness of the virus to adjust among its dynamic range of hosts and vectors. A mixture of coincidence and antagonism has also been reported previously in the case of HCV [31] and enterovirus 71 [13]. It was suggested that the coincident portions of codon usage among viruses and their hosts could enable the corresponding amino acids to be translated efficiently, while the antagonistic portions of codon usage may enable viral proteins to be folded properly, although the translation efficiency of the corresponding amino acids might decrease [31].

**Figure 2:**
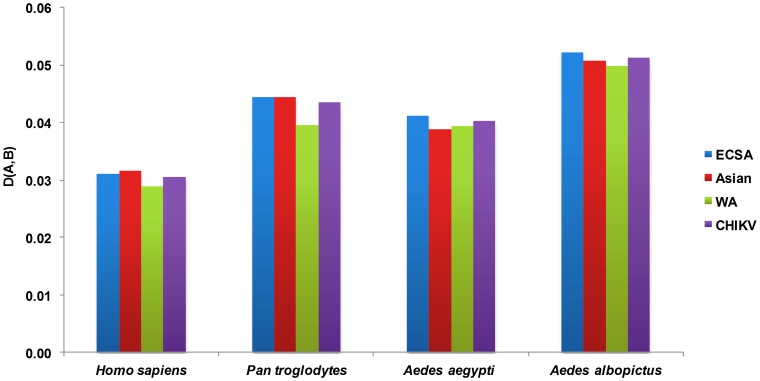
The similarity index analysis of the codon usage between CHIKV, its hosts and transmission vectors.

Although the comparative analysis of individual RSCU values as given above is frequently employed as a method of estimating the effect of synonymous codons usage of the hosts on that of specific viruses, it has its limitations in revealing the effect of the overall codon usage of the hosts on the formation of codon usage patterns of the viruses. Therefore, we took advantage of a method proposed recently that estimates the similarity degree of the overall codon usage patterns comprehensively between viruses and their hosts by treating the 59 synonymous codons as 59 different spatial vectors. The advantage of this formula, as reported by the authors in the case of dengue viruses, is that the comparative overall codon usage takes the place of the direct estimation of each synonymous codon usage; thus, the new method avoids the situation that the variations of 59 synonymous codon usage confuse the correct estimation of the effect of the host on the virus for codon usage [36]. The similarity index *D(A,B)* was therefore calculated for each genotype of CHIKV in relation to its hosts and vectors. The similarity index was found to be highest for *A. albopictus vs*. CHIKV group followed by *P. troglodytes vs*. CHIKV, *A. aegypti vs*. CHIKV and lowest in the case of *H. sapiens vs*. CHIKV (Figure 2), indicating that the effect of *A. albopictus* and *P. troglodytes* on the formation of the overall codon usage patterns of CHIKV is relatively higher than that of the *A. aegypti* and *H. sapiens*. Secondly, we computed the effect of transmission vectors on the formation of the overall codon usage patterns of three genotypes of CHIKV. *A. aegypti* had the strongest effect on the east central south African (ECSA) genotype, followed by West African (WA) and Asian genotypes. In the case of *A. albopictus*, the strongest effect was noted on the ECSA genotype, followed by Asian and WA genotypes. As for the effects of the two primates on the formation of the overall codon usage of CHIKV, the strongest effect of *H. sapiens* was on the Asian genotype, closely followed by the ECSA and WA genotypes. By contrast, *P. troglodytes* had its strongest and equal effect on ECSA and Asian genotypes, followed by WA genotype (Figure 2). Therefore, from the similarity index analysis, we observed that selection pressure from hosts and vectors have contributed to shaping the molecular evolution of CHIKV at the level of codon usage. The effect of the hosts was unevenly distributed among different genotypes, potentially indicating different evolutionary rates of CHIKV isolates. The calculation of the effects of primates and transmission vectors on the overall codon usage patterns of CHIKV showed that *P. troglodytes* and *A. albopictus* dominate the effects of *H. sapiens* and *A. aegypti*, respectively, on the formation of the overall codon usage patterns of CHIKV (Figure 2). The stronger effect of *P. troglodytes* than *H. sapiens* could also be attributed to the maintenance of CHIKV in a yellow fever-like zoonotic sylvatic cycle and its dependence upon non-human primates as reservoir hosts [7], [9]. Moreover, the similarity index of codon usage was also the highest between CHIKV and *A. albopictus*, as compared with *A. aegypti*, *P. troglodytes* and *H. sapiens*. The successful human-to-human transmission of CHIKV depends on *Aedes* mosquitoes [7], [9]; therefore, the stronger effect of *A. albopictus* on all three genotypes of CHIKV suggests that this vector might be a more efficient reservoir for viral replication and transmission compared with *A. aegypti*. These results are in agreement with recent studies showing more efficient dissemination and transmission of CHIKV by *A. albopictus*, which contribute to its ongoing re-emergence in a series of large-scale epidemics [37], [38].

### Trends of Codon Usage Variation in CHIKV

#### Correspondence Analysis (COA)

**Figure 3:**
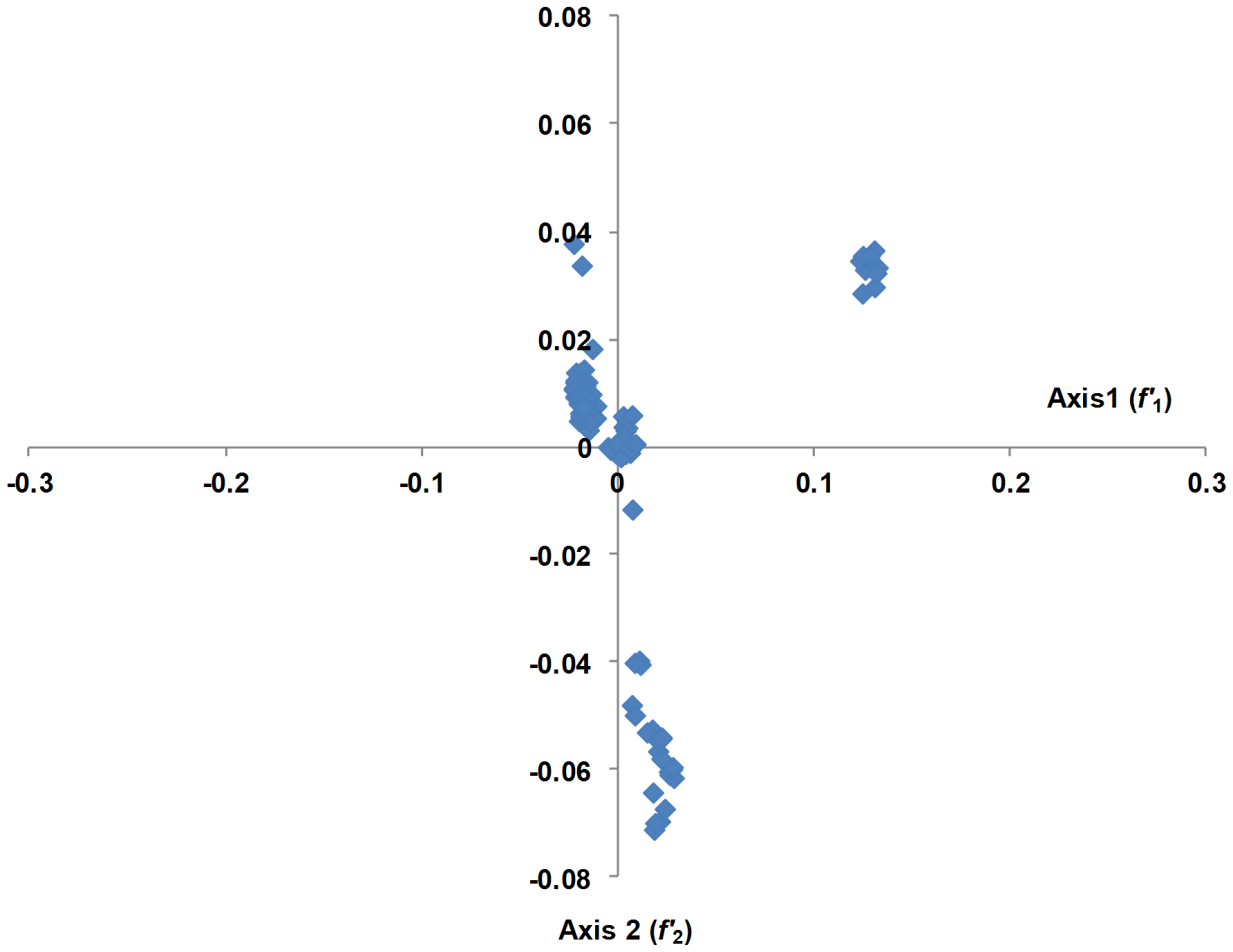
Correspondence analysis of codon usage patterns in CHIKV genomes.

**Figure 4:**
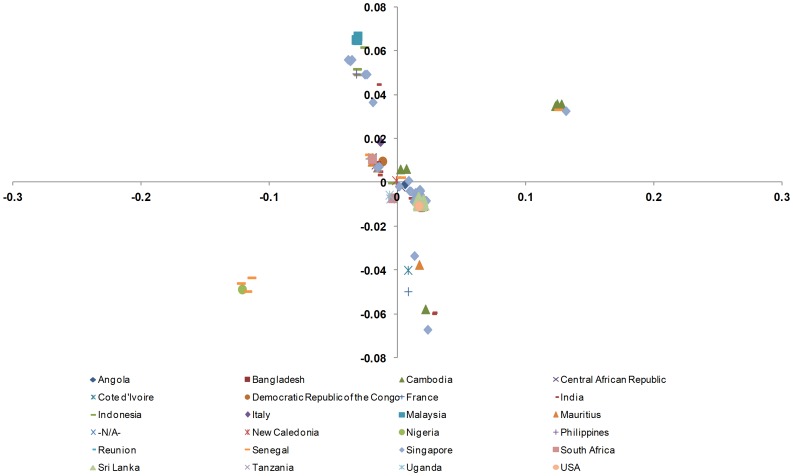
Correspondence analysis of codon usage patterns in CHIKV genomes based on region of isolation.

**Figure 5:**
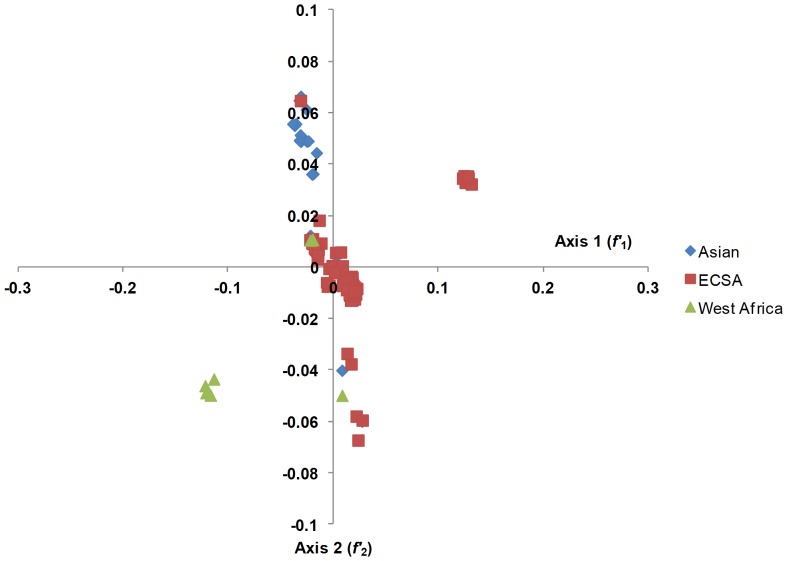
Correspondence analysis of codon usage patterns in CHIKV genomes based on virus genotypes.

Codon usage is multivariate by its very nature; therefore, it is necessary to analyze the data using multivariate statistical techniques, such as COA [39]. Therefore, to determine the trends in codon usage variation among different CHIKV genomes, we performed COA on the RSCU values, which were examined as a single dataset based on the RSCU value of each coding region (Figure 3). The first principal axis (*f*′_1_) accounted for 53.57% of the total variation, and the next three axes (*f*′_2_−*f*′_4_) accounted for 25.16%, 7.62%, and 2.06% of the total variation in synonymous codon usage, respectively. For further analysis, plots were reconstructed based on different geographical locations (Figure 4) and genotypes of CHIKV isolates (Figure 5). As expected the CHIKV isolates belonging to ECSA genotype were distributed across all planes of axes. When these plots were accessed on regional basis, it was found that different genotypes are circulating in single country. This analysis showed that the three different genotypes of CHIKV might have common ancestor. This further implies that the geographical diversity and associated factors, such as presence of favorable transmission vectors, climate features, host range and susceptibility, have also contributed to shaping the molecular evolution and codon usage in CHIKV, even though it appears to be less influential than mutational pressure (based on the current analysis).

#### Effect of mutational pressure in shaping the codon usage patterns in CHIKV

**Figure 6:**
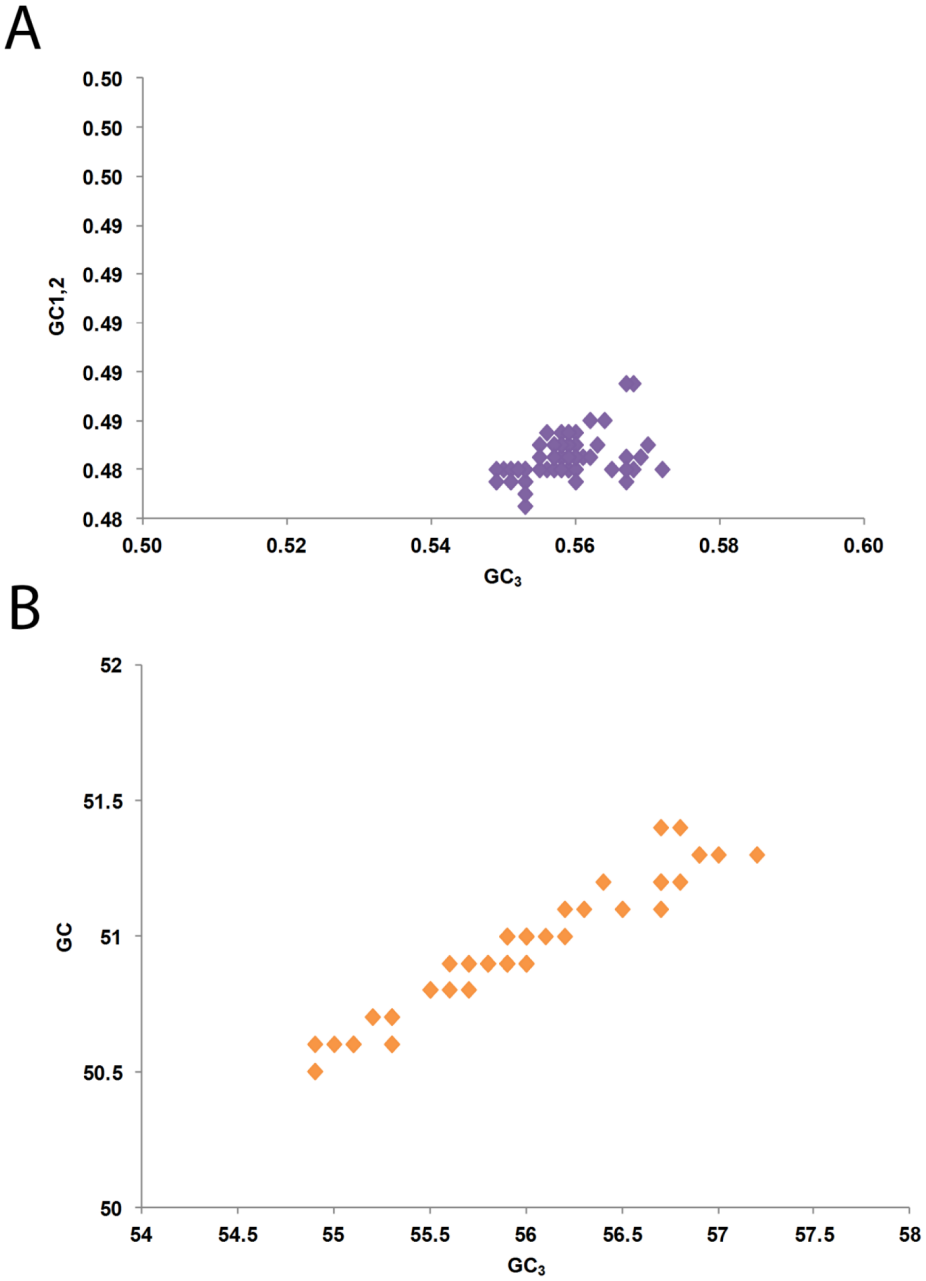
Correlation analysis. (A) GC_1,2_ with that at GC_3_, (B) GC with that at GC_3_.

Mutational pressure and natural selection are considered the two major factors that shape codon usage patterns [40]. A general mutational pressure, which affects the whole genome, would certainly account for the majority of the codon usage among certain RNA viruses [21]. To determine the extent of the influence of these two factors on CHIKV codon usage, we performed correlation analysis between different nucleotide constraints. A complex correlation was observed among different nucleotide constraints (Table 3). U_3_% had a significant positive correlation with U% (*r = *0.621, *P<*0.01) and G% (*r = *0.185, *P<*0.05), whereas it had significant negative correlations with C% (*r = *−0.606, *P<*0.01) A% (*r = *−0.278, *P<*0.01) and GC% (*r = *−0.806, *P<*0.01). C_3_% had significant positive correlation with C% (*r = *0.621, *P<*0.01), A (*r = *0.261, *P<*0.01) and GC% (*r = *0.798, *P<*0.01), and negative correlations with U% (*r = *−0.5877, *P<*0.01) and G% (*r = *−0.217, *P<*0.01). A_3_% had positive correlations with A (*r = *0.625, *P<*0.01), C% (*r = *0.327, *P<*0.01) and negative correlations with U% (*r = *−0.373, *P<*0.01) and G% (*r = *−0.576, *P<*0.01), whereas no correlation was observed between A_3_% and the GC%. G_3_% was positively correlated with G% (*r = *0.658, *P<*0.01) U% (*r = *0.354, *P<*0.01), and negatively correlated with C% (*r = *−0.377, *P<*0.01) and A% (*r = *−0.610, *P<*0.01); the correlation with the GC% was non-significant. In the case of GC_3_%, positive correlation was noted with C% (*r = *0.498, *P<*0.01) and GC% (*r = *0.852, *P<*0.01), and negative correlation with U% (*r = *−0.480, *P<*0.01); the correlation with G% was non-significant. Finally the GC and GC_12_ were also compared with GC_3_ and a highly significant positive correlations (*r = *0.28, *P<*0.01; GC_12_ versus GC_3_) (*r = *0.85, *P<*0.01; GC versus GC_3_) was observed as shown in Figure 6A and 6B respectively. Furthermore, a significant negative correlation between GC_3_ and ENC values was also observed (*r = *−0.756, *P<*0.01). This analysis collectively indicates that mutational pressure is most likely responsible for the patterns of nucleotide composition and, therefore, codon usage patterns, because the effects were present at all codon positions.

**Table 3:** Summary of correlation analysis between nucleotide constraints in CHIKV genomes.

	A_3_%	U_3_%	C_3_%	G_3_%	GC_3_%
**A%**	0.625**	−0.278**	0.261**	−0.610**	0.090^NS^
**U%**	−0.373**	0.621**	−0.587**	0.354**	−0.480**
**C%**	0.327**	−0.606**	0.621**	−0.377**	0.498**
**G%**	−0.576**	0.185*	−0.217**	0.658**	−0.080^NS^
**GC%**	0.103^NS^	−0.806**	0.798**	−0.153^NS^	0.852**

The numbers in the each column represents correlation coefficient “*r*” values, which are calculated in each correlation analysis.

NS: non-significant (*P*>0.05).

*represents 0.01<*P*<0.05.

**represents *P*<0.01.

**Figure 7:**
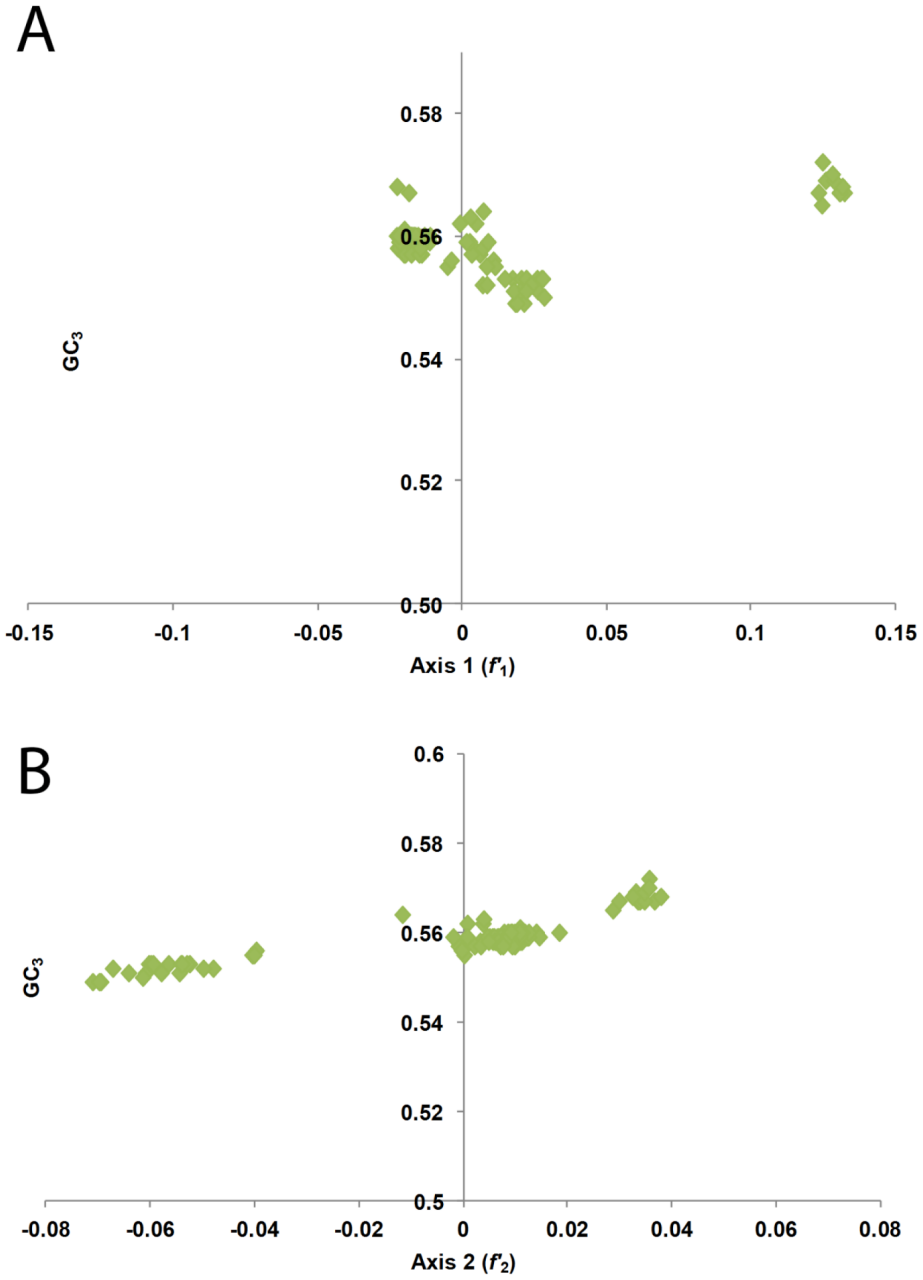
Correlation between the first axis (A) and second axis (B) values of COA and GC_3_ values.

In addition to correlation analysis, linear regression analysis was also performed to determine correlations between the first two principle axes (*f*′_1_ and *f*′_2_) and nucleotide constraints of CHIKV genomes. Again, several significant correlations were observed between the two principle axes and nucleotide contents (Table 4). *f*′_1_ showed a significantly positive correlation with U_3_% (*r* = 0.31, *P<*0.01), G_3_% (*r* = 0.58, *P<*0.01), U% (*r* = 0.25, *P<*0.01) and C% (*r* = 0.51, *P<*0.01); however, it showed significantly negative correlations with A% (*r* = −0.54, *P<*0.01), G% (*r* = −0.29, *P<*0.01), A_3_% (*r* = −0.50, *P<*0.01), C_3_ (*r* = −0.35, *P<*0.01), GC_3_ (*r* = −0.24, *P<*0.01; Figure 7A) and GC% (*r* = −0.21, *P<*0.01). In the case of *f_2_*, A_3_%, G_3_% and C% had non-significant correlations. *f*′_2_ axis showed significantly positive correlations with C_3_ (*r* = 0.69, *P<*0.01), GC_3_% (*r* = 0.74, *P<*0.01; Figure 7B), GC% (*r* = 0.64, *P<*0.01), A% (*r* = 0.17, *P<*0.05) and G% (*r* = 0.39, *P<*0.01) whereas, negative correlations with U_3_% (*r* = −0.66, *P<*0.01), and U% (*r* = −0.34, *P<*0.01) (Table 4). Our analysis shows that mutational pressure has played a major role in shaping the dynamics of codon usage patterns within CHIKV genomes.

**Table 4:** Summary of correlation between the first two principle axes and nucleotide constraints in CHIKV genomes.

Composition (%)	*f*′_1_ (53.57%)	*f*′_2_ (25.16%)
A_3_	−0.50**	−0.97^NS^
U_3_	0.310**	−0.659**
C_3_	−0.35**	0.69**
G_3_	0.58**	−0.134^NS^
GC_3_	−0.24**	0.740**
GC	−0.21*	0.640**
A	−0.54**	0.174*
U	0.25**	−0.340**
G	−0.29**	0.390**
C	0.517**	−0.126^NS^

The numbers in the each column represents correlation coefficient “*r*” values, which are calculated in each correlation analysis.

NS: non-significant (*P*>0.05).

*represents 0.01<*P*<0.05.

**represents *P*<0.01.

#### Correlation analysis between ENC and GC_3_ values

**Figure 8:**
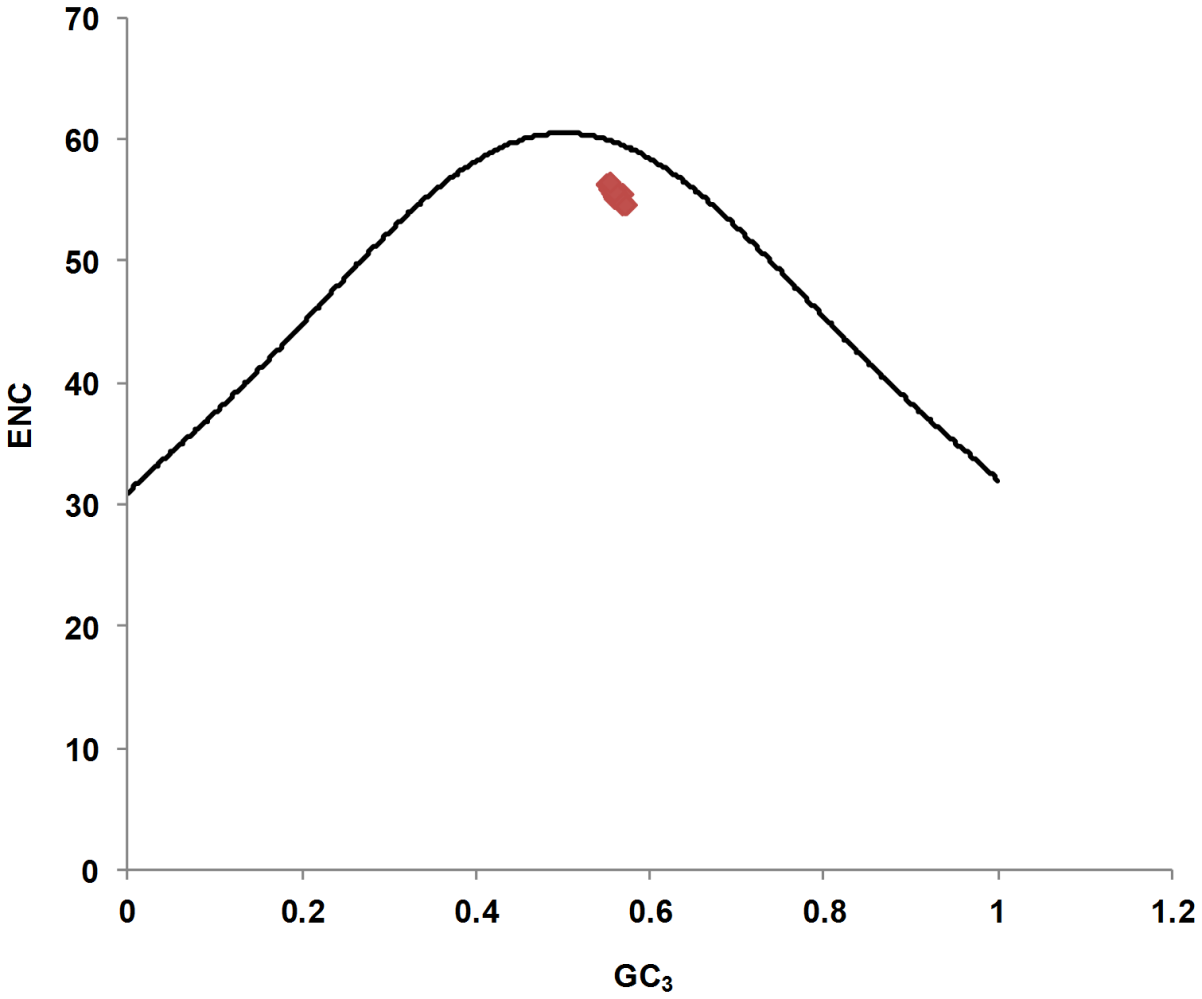
The relationship between the effective number of codons (ENC) values and the GC content at the third synonymous codon position (GC_3_). The curve indicates the expected codon usage if GC compositional constraints alone account for codon usage bias.

A plot of ENC versus GC_3_ (Nc plot) is widely used to study codon usage variation among genes in different organisms. It has been postulated that an ENC-plot of genes, whose codon choice is constrained only by a G_3_+ C_3_ mutational bias, will lie on or just below the continuous curve of the predicted ENC values [30]. Although, the nucleotide composition correlation analysis showed that codon usage in CHIKV genomes is mainly caused by compositional constraints or mutational pressure, we were interested to determine the possible influence of other factors, such as natural selection. Therefore, we constructed a corresponding relation distribution plot between the ENC and GC_3_ values. As shown in Figure 8, all points aggregated closely towards the right side under the expected ENC curve, indicating that, apart from mutation pressure, the codon usage patterns have also been influenced by other factors to some extent.

#### Relationship between dinucleotide and codon usage patterns in CHIKV

It has been suggested that dinucleotide bias can affect overall codon usage bias in several organisms, including DNA and RNA viruses [41]–[43]. To study the possible effect of dinucleotides on codon usage in CHIKV genomes, we calculated the relative abundances of the 16 dinucleotides from the coding sequences of CHIKV. The occurrences of dinucleotides were not randomly distributed, and no dinucleotides were present at the expected frequencies (Table 5). Under-representation of CpG dinucleotides in different RNA and DNA viruses has been reported [41]. In the case of CHIKV, the relative abundance of CpG showed deviation from the “normal range” (mean ± SD = 0.808±0.016) and was under-represented. Interestingly, GpC dinucleotides also deviated from the normal range and were instead slightly over-represented (mean ± SD = 1.001±0.007) (Table 5). The RSCU values of the eight codons containing CpG (CCG, GCG, UCG, ACG, CGC, CGG, CGU, and CGA) and the six codons containing GpC (GCU, GCC, GCA, UGC, AGC, GGC) were also analyzed to determine the possible effects of CpG and GpC representations on codon usage bias. In the case of CpG-containing codons, all codons were under-represented (RSCU<1.6) and were not preferred codons for their respective amino acid, except for CCG (RSCU = 1.26), a preferred codon for proline (Table 2). On the other hand, despite slight over-representation of the GpC dinucleotide, all GpC-containing codons were also under-represented (RSCU<1.6) and were not preferred codons for their respective amino acids, with two exceptions; GCA (Ala, RSCU = 1.43) and UGC (Cys, RSCU = 1.33) (Table 2). It has been proposed that CpG deficiency in pathogens is associated with the immunostimulatory properties of unmethylated CpGs, which are recognized by the host’s innate immune system as a pathogen signature [28]. Recognition of umethylated CpGs by Toll like receptor 9 (TLR9), a type of intracellular pattern recognition receptor (PRR), leads to activation of several immune response pathways [44]. The vertebrate immune system relies on unmethylated CpG recognition in DNA molecules as a signature of infection, and CpG under-representation in RNA viruses is exclusively observed in vertebrate viruses; therefore, it is reasonable to suggest that a TLR9-like mechanism exists in the vertebrate immune system that recognizes CpGs when in an RNA context (such as in the genomes of RNA viruses) and triggers immune responses [45].

**Table 5:** Summary of correlation analysis between the first two principal axes and relative abundance of dinucleotides in CHIKV genomes.

		UU	UC	UA	UG	CU	CC	CA	CG
**Mean ± SD**		0.954±0.030	0.935±0.020	0.859±0.022	1.275±0.022	1.082±0.026	0.979±0.017	1.125±0.017	0.808±0.016
**Range**		0.886–1.082	0.862–0.964	0.784–0.934	1.214–1.329	1.022–1.107	0.946–1.025	1.058–1.172	0.781–0.856
**Axis 1**	***r***	0.755**	−0.664**	0.213*	−0.071^NS^	−0.724**	0.612**	−0.262**	0.236**
	***P***	0.000	0.000	0.011	0.403	0.000	0.000	0.002	0.005
**Axis 2**	***r***	−0.357**	0.233**	−0.665**	0.429**	0.418**	−0.305**	0.611**	−0.548**
	***P***	0.000	0.005	0.000	0.000	0.000	0.000	0.000	0.000
		**AU**	**AC**	**AA**	**AG**	**GU**	**GC**	**GA**	**GG**
**Mean ± SD**		0.929±0.015	1.055±0.014	0.987±.0078	1.024±0.006	1.054±0.014	1.001±0.007	1.012±0.009	0.931±0.010
**Range**		0.884–0.987	0.998–1.097	0.963–1.008	1.009–1.037	1.007–1.117	0.965–1.020	0.996–1.037	0.900–0.954
**Axis 1**	***r***	0.145^NS^	0.39^NS^	−0.387**	0.236**	0.80^NS^	−0.009^NS^	0.698**	−0.366**
	***P***	0.086	0.645	0.000	0.005	0.345	0.919	0.000	0.000
**Axis 2**	***r***	−0.601**	−0.381**	0.221**	−0.404**	−0.508**	0.279**	−0.288**	0.168*
	***P***	0.000	0.000	0.009	0.000	0.000	0.001	0.001	0.047

NS: non-significant (*P*>0.05).

*represents 0.01<*P*<0.05.

**represents *P*<0.01.

Compared with differential (over- and under-) representation of CpGs in different organisms, UpA under-representation also exists in several organisms, including vertebrates, invertebrates, plants and prokaryotes [41]. The presence of TpA in two out of three canonical stop codons and in transcriptional regulatory motifs (e.g., the TATA box sequence) is believed to be responsible for its under-representation. Therefore, UpA under-representation is expected to reduce the risk of nonsense mutations and minimizes improper transcription [43], [46]. In the case of CHIKV, the relative abundance of UpA also deviated from the “normal range” (mean ± SD = 0.859±0.022) and was under-represented, similarly to CpG. The six codons containing UpA (UUA, CUA, GUA, UAU, UAC and AUA) were also under-represented (RSCU<1.6) and were not preferred codons for their respective amino acids. The CpA (mean ± SD = 1.125±0.017) and UpG (mean ± SD = 1.275±0.022) dinucleotides were over-represented compared with the rest of the 14 dinucleotide pairs (Table 5). Similarly, the eight codons containing CpA (UCA, CCA, ACA, GCA, CAA, CAG, CAU and CAC) and five codons containing UpG (UUG, CUG, GUG, UGU and UGC) were also over-represented compared with the rest of the codons for their respective amino acids and a majority of them were also preferential codons for their respective amino acids, based on RSCU analysis (Table 2). Over-representation of CpA and UpG in different organisms has been observed and is regarded as a consequence of the under-representation of CpG dinucleotides. One possible explanation is that methylated cytosines are prone to mutate into thymines through spontaneous deamination, resulting in the dinucleotide TpG and the subsequent presence of a CpA on the opposite strand after DNA replication [47]. However, this theory cannot explain under-representation of CpGs in RNA viruses. Moreover, under-representation of CpGs has also been observed in several vertebrate viruses, where it is independent of their genomic composition and replication cycles. Recently, two studies performed large-scale dinucleotide analyses in different viruses and suggested that the CpG usage of +ssRNA viruses is affected greatly by their hosts. As a result, most +ssRNA viruses mimic their hosts’ CpG usage and the existence of an RNA dinucleotide recognition system, probably linked to the innate immune system of the host, has also been proposed [41], [48].

Finally, the relative abundance of dinucleotides was also correlated with the first two principal axes. Among the 16 dinucleotides, 11 significantly (positive and negative) correlated with the first axis and 16 significantly (positive and negative) correlated with the second axis (Table 5). These observations indicated that the composition of dinucleotides determines the variation in synonymous codon usage. Therefore, from the present dinucleotide composition analysis, it is evident that selection pressure associated with (i) maintenance of efficient replication and transmission cycles among multiple hosts, and (ii) evolution of escape mechanisms to evade from the host antiviral responses, have contributed to shaping the overall synonymous codon usage in CHIKV.

#### Effect of natural selection in shaping the codon usage patterns in CHIKV

It has been suggested that if synonymous codon usage bias is affected by mutational pressure alone, then the frequency of nucleotides A and U/T should be equal to that of C and G at the synonymous codon third position [26]. However, in case of CHIKV genomes, variations in nucleotide base compositions were noted (Table 1), indicating that other factors, such as natural selection, could also influence overall synonymous codon usage bias. As the role of natural selection is also evident from previous codon usage analysis studies in several viruses [25], [26], [49], we were interested to determine to what extent natural selection might be involved in the codon usage patterns of CHIKV. For this purpose, we computed the GRAVY and aromaticity (ARO) values for each CHIKV isolate (Table S1) and a linear regression analysis was performed between GRAVY, ARO and the *f*′_1_, *f*′_2_, ENC, GC and GC_3_ values. The analysis results showed that the GRAVY values were not significant for *f*′_1_ and were highly significant for *f*′_2_, ENC, GC_3_ and GC. In the case of ARO, an opposite trend was observed: ARO values were significantly negatively correlated with *f*′_1_ and correlations with *f*′_2_, ENC, GC_3_ and GC were not significant (Table 6). These results indicated that, although natural selection has influenced codon usage of CHIKV genomes to some extent, it is much weaker compared with mutational pressure.

**Table 6:** Correlation analysis among GRAVY, ARO, ENC, GC_3_, GC and the first two principle axes.

		*f*′_1_ (53.57%)	*f*′_2_ (25.16%)	ENC	GC_3_	GC
GRAVY	*r*	0.118^NS^	−0.558**	0.420**	−0.529**	−0.568**
	*P*	0.164	0.000	0.003	0.000	0.000
ARO	*r*	0.169*	−0.149^NS^	0.081^NS^	0.026^NS^	−0.021^NS^
	*P*	0.045	0.077	0.340	0.758	0.803

ARO: Aromaticity.

NS: non-significant (*P*>0.05).

*represents 0.01<*P*<0.05.

**represents *P*<0.01.

## Conclusions

Taken together, our analysis showed that overall codon usage bias in CHIKV is slightly biased, and the major factor that has contributed to shaping codon usage pressure is mutational pressure. In addition, contributions of other factors, including hosts, geography, dinucleotides composition and natural selection, are also evident from our analysis. Our data suggested that codon usage in CHIKV is undergoing an evolutionary process, probably reflecting a dynamic process of mutation and natural selection to re-adapt its codon usage to different environments and hosts. To the best our knowledge, this is first report of codon usage analysis in CHIKV and is expected to deepen our understanding of the mechanisms contributing towards codon usage and evolution of CHIKV.

## Materials and Methods

### Sequences

The complete genome sequences of 141 CHIKV isolates (in FASTA format) were obtained from the National Center for Biotechnology (NCBI) GenBank database (http://www.ncbi.nlm.nih.gov). The accession numbers and other detailed information of the selected CHIKVs’ genomes, such as isolation date, isolation place, host and genome size were also retrieved (Table 7).

**Table 7:** Demographics of CHIKV genomes analyzed in present study.

No	Strain Name	GenBank Accession	Length (bp)	Year	Host	Country	Genotype
1	Ross low-psg	HM045811	11775	1953	Human	Tanzania	ECSA
2	Vereeniging	HM045792	11836	1956	Human	South Africa	ECSA
3	TH35	HM045810	11986	1958	Human	Thailand	Asian
4	LSFS	HM045809	11753	1960	Human	DRC	ECSA
5	Angola M2022	HM045823	11754	1962	–	Angola	ECSA
6	A301	HM045821	11823	1963	Bat	Senegal	ECSA
7	Gibbs 63–263	HM045813	11976	1963	Human	India	Asian
8	I-634029	HM045803	11897	1963	Human	India	Asian
9	IND-63-WB1	EF027140	11784	1963	–	India	Asian
10	IbH35	HM045786	11844	1964	Human	Nigeria	WA
11	PM2951	HM045785	11844	1966	Mosquito	Senegal	WA
12	SH 3013	HM045816	11823	1966	Human	Senegal	WA
13	PO731460	HM045788	11988	1973	Human	India	Asian
14	IND-73-MH5	EF027141	11805	1973	–	India	Asian
15	1455–75	HM045814	11939	1975	Human	Thailand	Asian
16	AR 18211	HM045805	11686	1976	Mosquito	South Africa	ECSA
17	3412–78	HM045808	11968	1978	Human	Thailand	Asian
18	HB78	HM045822	11753	1978	Human	CAR	ECSA
19	ArD 30237	HM045815	11823	1979	Mosquito	Senegal	WA
20	ArA 2657	HM045818	11823	1981	Mosquito	Cote d’Ivoire	WA
21	IPD/A SH 2807	HM045804	11847	–	Human	Senegal	WA
22	UgAg4155	HM045812	11774	1982	Human	Uganda	ECSA
23	JKT23574	HM045791	11992	1983	Human	Indonesia	Asian
24	37997	AY726732	11881	1983	Mosquito	Senegal	WA
25	DakAr B 16878	HM045784	11772	1984	Mosquito	CAR	ECSA
26	RSU1	HM045797	11979	1985	Human	Indonesia	Asian
27	Hu/85/NR/001	HM045800	11897	1985	Human	Philippines	Asian
28	PhH15483	HM045790	11907	1985	Human	Philippines	Asian
29	ALSA-1	HM045806	11768	1986	–	India	ECSA
30	CAR256	HM045793	11767	–	–	CAR	ECSA
31	6441–88	HM045789	11855	1988	Human	Thailand	Asian
32	ArD 93229	HM045819	11860	1993	Mosquito	Senegal	WA
33	ArA 30548	HM045820	11817	1993	Mosquito	Cote d’Ivoire	WA
34	CO392-95	HM045796	11979	1995	Human	Thailand	Asian
35	SV0444-95	HM045787	11968	1995	Human	Thailand	Asian
36	K0146-95	HM045802	11975	1995	–	Thailand	Asian
37	IND-00-MH4	EF027139	11814	2000	Human	India	ECSA
38	HD 180760	HM045817	11832	2005	Human	Senegal	WA
39	IMTSSA6424C	FR717337	11559	2005	Human	France	ECSA
40	IMTSSA6424S	FR717336	11559	2005	Human	France	ECSA
41	BNI-CHIKV_899	FJ959103	11832	2006	Human	Mauritius	ECSA
42	MY019IMR/06/BP	EU703761	12028	2006	Human	Malaysia	Asian
43	DHS4263-Calif AB	HM045794	11774	2006	Human	USA	ECSA
44	MY003IMR/06/BP	EU703760	12028	2006	Human	Malaysia	Asian
45	MY002IMR/06/BP	EU703759	12028	2006	Human	Malaysia	Asian
46	DRDE-06	EF210157	11774	2006	Human	India	ECSA
47	0611aTw	FJ807896	11811	2006	Human	Singapore	ECSA
48	TM25	EU564334	11772	2006	Human	Mauritius	ECSA
49	IND-KA51	FJ000068	11812	2006	Human	India	ECSA
50	IND-MH51	FJ000067	11812	2006	Human	India	ECSA
51	IND-GJ52	FJ000062	11812	2006	Human	India	ECSA
52	IND-GJ53	FJ000065	11813	2006	Human	India	ECSA
53	IND-KR51	FJ000066	11812	2006	Human	India	ECSA
54	IND-GJ51	FJ000064	11807	2006	Human	India	ECSA
55	IND-06-Guj	JF274082	11829	2006	Human	India	ECSA
56	IND-KA52	FJ000063	11812	2006	Human	India	ECSA
57	RGCB05/KL06	GQ428211	11764	2006	Human	India	ECSA
58	RGCB03/KL06	GQ428210	11764	2006	Human	India	ECSA
59	CHIK31	EU564335	11810	2006	Human	India	ECSA
60	SL10571	AB455494	11829	2006	Human	–	ECSA
61	SL11131	AB455493	11829	2006	Human	–	ECSA
62	IND-06-KA15	EF027135	11729	2006	Human	India	ECSA
63	D570/06	EF012359	11806	2006	–	Mauritius	ECSA
64	IND-06-RJ1	EF027137	11767	2006	–	India	ECSA
65	IND-06-AP3	EF027134	11779	2006	Human	India	ECSA
66	IND-06-TN1	EF027138	11750	2006	Human	India	ECSA
67	LR2006_OPY1	DQ443544	11840	2006	Human	Reunion	ECSA
68	IND-06-MH2	EF027136	11800	2006	Human	India	ECSA
69	SL-CR 3	HM045799	11758	2007	Human	Sri Lanka	ECSA
70	ITA07-RA1	EU244823	11788	2007	–	Italy	ECSA
71	SL-CK1	HM045801	11766	2007	Human	Sri Lanka	ECSA
72	0706aTw	FJ807897	12013	2007	Human	Indonesia	Asian
73	LKRGCH1507	FJ445428	11717	2007	Human	Sri Lanka	ECSA
74	IND-KR52	FJ000069	11812	2007	Human	India	ECSA
75	DRDE-07	EU372006	11774	2007	Human	India	ECSA
76	LKMTCH2707	FJ445427	11717	2007	Human	Sri Lanka	ECSA
77	RGCB80/KL07	GQ428212	11764	2007	Human	India	ECSA
78	RGCB120/KL07	GQ428213	11764	2007	Human	India	ECSA
79	0810aTw	FJ807898	11811	2008	Human	Bangladesh	ECSA
80	SD08Pan	GU199351	11793	2008	Human	China	ECSA
81	0810bTw	FJ807899	11811	2008	Human	Malaysia	ECSA
82	SGEHICHS277108	FJ445510	11800	2008	Human	Singapore	ECSA
83	SVUKDP-08	JN558835	11733	2008	Human	India	ECSA
84	FD080178	GU199352	11677	2008	Human	China	ECSA
85	FD080008	GU199350	11687	2008	Human	China	ECSA
86	FD080231	GU199353	11687	2008	Human	China	ECSA
87	SGEHICHD13508	FJ445511	11719	2008	Human	Singapore	ECSA
88	LK(PB)CH5808	FJ513637	11710	2008	Human	Sri Lanka	ECSA
89	LK(PB)CH3008	FJ513632	11693	2008	Human	Sri Lanka	ECSA
90	LK(PB)CH1608	FJ513629	11716	2008	Human	Sri Lanka	ECSA
91	LK(PB)CH5308	FJ513635	11726	2008	Human	Sri Lanka	ECSA
92	LK(PB)chik6008	GU013529	11718	2008	Human	Sri Lanka	ECSA
93	LK(PB)CH1008	FJ513628	11722	2008	Human	Sri Lanka	ECSA
94	LK(PB)chik3408	GU013528	11715	2008	Human	Sri Lanka	ECSA
95	LK(EH)CH6708	FJ513654	11717	2008	Human	Sri Lanka	ECSA
96	LK(EH)CH7708	FJ513657	11696	2008	Human	Sri Lanka	ECSA
97	LK(EH)CH4408	FJ513645	11714	2008	Human	Sri Lanka	ECSA
98	LK(EH)CH20108	FJ513679	11717	2008	Human	Sri Lanka	ECSA
99	LK(EH)CH18608	FJ513675	11716	2008	Human	Sri Lanka	ECSA
100	LK(EH)chik19708	GU013530	11714	2008	Human	Sri Lanka	ECSA
101	LKEHCH13908	FJ445426	11717	2008	Human	Sri Lanka	ECSA
102	LK(EH)CH17708	FJ513673	11710	2008	Human	Sri Lanka	ECSA
103	SGEHICHT077808	FJ445484	11790	2008	Human	Singapore	ECSA
104	RGCB356/KL08	GQ428215	11764	2008	Human	India	ECSA
105	RGCB355/KL08	GQ428214	11764	2008	Human	India	ECSA
106	SGEHICHS422308	FJ445432	11722	2008	Human	Singapore	ECSA
107	SGEHICHS421708	FJ445431	11722	2008	Human	Singapore	ECSA
108	SGEHICHD93508	FJ445430	11722	2008	Human	Singapore	ECSA
109	SGEHICHD96808	FJ445463	11729	2008	Human	Singapore	ECSA
110	SGEHICHS424108	FJ445443	11714	2008	Human	Singapore	ECSA
111	SGEHICHS422808	FJ445433	11729	2008	Human	Singapore	ECSA
112	SGEHICHS425208	FJ445445	11719	2008	Human	Singapore	ECSA
113	SGEHICHD122508	FJ445502	11717	2008	Human	Singapore	ECSA
114	CU-Chik10	GU301780	11811	2008	Human	Thailand	ECSA
115	SVUCTR-09	JN558834	11733	2009	Human	India	ECSA
116	SVUKDP-09	JN558836	11733	2009	Human	India	ECSA
117	CU-Chik661	GQ905863	11752	2009	Human	Thailand	ECSA
118	CU-Chik683	GU301781	11811	2009	Human	Thailand	ECSA
119	CU-Chik_OBF	GU908223	11670	2009	Mosquito	Thailand	ECSA
120	CU-Chik009	GU301779	11811	2009	Human	Thailand	ECSA
121	NL10/152	KC862329	11836	2010	Human	Indonesia	ECSA
122	GD05/2010	JX088705	11811	2010	Human	China	ECSA
123	GZ0991	JQ065890	11684	2010	Human	China	ECSA
124	GD113	HQ846357	11720	2010	Human	China	ECSA
125	GD139	HQ846358	11730	2010	Human	China	ECSA
126	GD115	HQ846356	11746	2010	Human	China	ECSA
127	GD134	HQ846359	11725	2010	Human	China	ECSA
128	GZ1029	JQ065891	11687	2010	Human	China	ECSA
129	CHI2010	JQ067624	11724	2010	Human	China	ECSA
130	NC/2011-568	HE806461	11621	2011	Human	New Caledonia	ECSA
131	V0603310_KH11_BTB	JQ861260	11743	2011	Human	Cambodia	ECSA
132	V1024311_KH11_PVH	JQ861256	11754	2011	Human	Cambodia	ECSA
133	V1024308_KH11_PVH	JQ861254	11750	2011	Human	Cambodia	ECSA
134	V1024314_KH11_PVH	JQ861258	11733	2011	Human	Cambodia	ECSA
135	V1024306_KH11_PVH	JQ861253	11745	2011	Human	Cambodia	ECSA
136	V1024310_KH11_PVH	JQ861255	11736	2011	Human	Cambodia	ECSA
137	V1024313_KH11_PVH	JQ861257	11755	2011	Human	Cambodia	ECSA
138	CHIKV-JC2012	KC488650	11889	2012	Human	China	Asian
139	Chik-sy	KF318729	12017	2012	Human	China	Asian
140	Wuerzburg	EU037962	11805	–	Human	Mauritius	ECSA
141	S27-African prototype	AF369024	11826	–	Human	–	ECSA

Dashes (−) indicates data not available. East Central South African, ECSA; Democratic Republic of Congo, DRC; Central African Republic, CAR; West African; WA.

### Compositional Analysis

The following compositional properties were calculated for the CHIKV genomes; (i) the overall frequency of occurrence of the nucleotides (A %, C %, U/T %, and G %); (ii) the frequency of each nucleotide at the third site of the synonymous codons (A_3%_, C_3%_, U_3%_ and G_3%_); (iii) the frequencies of occurrence of nucleotides G+C at the first (GC_1_), second (GC_2_), and third synonymous codon positions (GC_3_); (iv) the mean frequencies of nucleotide G+C at the first and the second position (GC_1,2_); and (v) the overall GC and AU content. The codons AUG and UGG are the only codons for Met and Trp, respectively, and the termination codons UAA, UAG and UGA do not encode any amino acids. Therefore, these five codons are expected not to exhibit any usage bias and were therefore excluded from the analysis.

### RSCU Analysis

The RSCU values for all the coding sequences of CHIKV genomes were calculated to determine the characteristics of synonymous codon usage without the confounding influence of amino acid composition and the size of coding sequence of different gene samples, following a previously described method [18]. The RSCU index was calculated as follows:
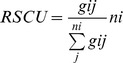
where *g_ij_* is the observed number of the *i*th codon for the *j*th amino acid which has *n_i_* kinds of synonymous codons. RSCU values represent the ratio between the observed usage frequency of one codon in a gene sample and the expected usage frequency in the synonymous codon family given that all codons for the particular amino acid are used equally. The synonymous codons with RSCU values >1.0 have positive codon usage bias and were defined as abundant codons, while those with RSCU values <1.0 have negative codon usage bias and were defined as less-abundant codons. When the RSCU values is 1.0, it means there is no codon usage bias for that amino acid and the codons are chosen equally or randomly [50]. Moreover, the synonymous codons with RSCU values >1.6 and <0.6 were treated as over-represented and under-represented codons, respectively [23].

### Influence of Overall Codon Usage of the Hosts on that of CHIKV

For the comparative analysis of codon usage between CHIKVs and its vectors and hosts; codon usage data for two transmission vectors (*A. aegypti*, *A. albopictus*), and hosts (*H. sapiens, P. troglodytes*) were obtained from the codon usage database (http://www.kazusa.or.jp/codon/) [51]. Zhou et al. proposed a method recently to determine the potential impact of the overall codon usage patterns of the hosts in the formation of the overall codon usage of viruses [36]. Here, we applied the same approach in case of CHIKV and the similarity index *D(A,B)* was calculated as follows:
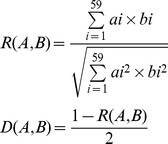
where *R(A,B)* is defined as a cosine value of an included angle between A and B spatial vectors representing the degree of similarity between CHIKV and a specific host at the aspect of the overall codon usage pattern, *a_i_* is defined as the RSCU value for a specific codon among 59 synonymous codons of CHIKV coding sequence, *b_i_* is termed as the RSCU value for the same codon of the host. *D(A,B)* represents the potential effect of the overall codon usage of the host on that of CHIKV, and its value ranges from zero to 1.0 [36].

### Measures of Relative Dinucleotides Abundance

The relative abundance of dinucleotides in the coding regions of CHIKV genomes was calculated using a previously described method [43]. A comparison of actual and expected dinucleotide frequencies of the 16 dinucleotides in coding regions of the CHIKV was also undertaken. The odds ratio was calculated using the following formula:
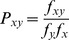
where *f*_x_ denotes the frequency of the nucleotide X, *f*_y_ denotes the frequency of the nucleotide Y, *f*_y_ *f*_x_ the expected frequency of the dinucleotide XY and *f*_xy_ the frequency of the dinucleotide XY, etc,. for each dinucleotide were calculated. As a conservative criterion, for *Pxy*>1.23 (or <0.78), the XY pair is considered to be over-represented (or under-represented) in terms of relative abundance compared with a random association of mononucleotides.

### CAI Analysis

The CAI is used as a quantitative method of predicting the expression level of a gene based on its codon sequence. The CAI value ranges from 0 to 1. The most frequent codons simply have the highest relative adaptiveness values, and sequences with higher CAIs are preferred over those with lower CAIs [32].

### ENC Analysis

The ENC is used to quantify the absolute codon usage bias of the gene (s) of interest, irrespective of gene length and the number of amino acids [30]. In this study, this measure was calculated to evaluate the degree of codon usage bias exhibited by the coding sequences of CHIKVs. The ENC values ranged from 20 for a gene showing extreme codon usage bias using only one of the possible synonymous codons for the corresponding amino acid, to 61 for a gene showing no bias using all possible synonymous codons equally for the corresponding amino acid. The larger the extent of codon preference in a gene, the smaller the ENC value is. It is also generally accepted that genes have a significant codon bias when the ENC value is less than or equal to 35 [30], [52]. The ENC was calculated using the following formula:
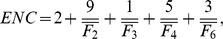
Where 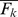
(k = 2,3,4,6) is the mean of 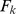
values for the *k*-fold degenerate amino acids, which is estimated using the formula as follows:
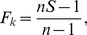
where *n* is the total number of occurrences of the codons for that amino acid and
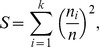
where *n_i_* is the total number of occurrences of the *i* th codon for that amino acid. Genes, whose codon choice is constrained only by a mutation bias, will lie on or just below the curve of the expected ENC values. Therefore, for elucidating the relationship between GC_3_ and ENC values, the expected ENC values for different GC_3_ were calculated as follows:
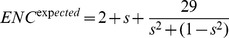
where *s* represents the given GC_3_% value [30].

### COA of Codon Usage

COA is a multivariate statistical method that is used to explore the relationships between variables and samples. In the present study, COA was used to analyze the major trends in codon usage patterns among CHIKVs coding sequences. COA involves a mathematical procedure that transforms some correlated variable (RSCU values) into a smaller number of uncorrelated variables called principal components. To minimize the effect of amino acid composition on codon usage, each coding sequence was represented as a 59 dimensional vector, and each dimension corresponded to the RSCU value of each sense codon, which only included several synonymous codons for a particular amino acid, excluding the codons AUG, UGG and the three stop codons.

### Correlation Analysis

Correlation analysis was carried out to identify the relationship between nucleotide composition and synonymous codon usage patterns of CHIKV. This analysis was implemented based on the Spearman’s rank correlation analysis. All statistical processes were carried out using the statistical software SPSS 16.0 for windows.

## Supporting Information

Table S1 Hydrophobicity (GRAVY) and aromaticity (ARO) indices in CHIKV genomes.
(DOCX)
