# Supplementary material for: Genome-Wide Analysis of Codon Usage and Influencing Factors in Chikungunya Viruses
Source: PLoS One. 2014 Mar 4;9(3):e90905. doi: 10.1371/journal.pone.0090905 (PMC3942501; doi:10.1371/journal.pone.0090905)
Supplement: Table S1 — Hydrophobicity (GRAVY) and aromaticity (ARO) indices in CHIKV genomes. (DOCX) [file pone.0090905.s001.docx]

**Table S1.** Hydrophobicity (GRAVY) and aromaticity (ARO) indices in CHIKV genomes.

| **No** | **ARO** | **GRAVY** |
| --- | --- | --- |
| 1 | 0.08 | -0.29 |
| 2 | 0.08 | -0.29 |
| 3 | 0.08 | -0.29 |
| 4 | 0.08 | -0.29 |
| 5 | 0.08 | -0.30 |
| 6 | 0.08 | -0.28 |
| 7 | 0.08 | -0.29 |
| 8 | 0.08 | -0.29 |
| 9 | 0.08 | -0.29 |
| 10 | 0.08 | -0.30 |
| 11 | 0.08 | -0.30 |
| 12 | 0.08 | -0.30 |
| 13 | 0.08 | -0.29 |
| 14 | 0.08 | -0.29 |
| 15 | 0.08 | -0.29 |
| 16 | 0.08 | -0.30 |
| 17 | 0.08 | -0.29 |
| 18 | 0.08 | -0.30 |
| 19 | 0.08 | -0.30 |
| 20 | 0.08 | -0.30 |
| 21 | 0.08 | -0.30 |
| 22 | 0.08 | -0.30 |
| 23 | 0.08 | -0.29 |
| 24 | 0.08 | -0.31 |
| 25 | 0.08 | -0.30 |
| 26 | 0.08 | -0.29 |
| 27 | 0.08 | -0.29 |
| 28 | 0.08 | -0.29 |
| 29 | 0.08 | -0.29 |
| 30 | 0.08 | -0.29 |
| 31 | 0.08 | -0.29 |
| 32 | 0.08 | -0.30 |
| 33 | 0.08 | -0.30 |
| 34 | 0.08 | -0.29 |
| 35 | 0.08 | -0.29 |
| 36 | 0.08 | -0.29 |
| 37 | 0.08 | -0.30 |
| 38 | 0.08 | -0.30 |
| 39 | 0.08 | -0.30 |
| 40 | 0.08 | -0.30 |
| 41 | 0.08 | -0.31 |
| 42 | 0.08 | -0.28 |
| 43 | 0.08 | -0.30 |
| 44 | 0.08 | -0.28 |
| 45 | 0.08 | -0.28 |
| 46 | 0.08 | -0.30 |
| 47 | 0.08 | -0.30 |
| 48 | 0.08 | -0.29 |
| 49 | 0.08 | -0.30 |
| 50 | 0.08 | -0.30 |
| 51 | 0.08 | -0.30 |
| 52 | 0.08 | -0.30 |
| 53 | 0.08 | -0.29 |
| 54 | 0.08 | -0.29 |
| 55 | 0.08 | -0.30 |
| 56 | 0.08 | -0.29 |
| 57 | 0.08 | -0.30 |
| 58 | 0.08 | -0.30 |
| 59 | 0.08 | -0.30 |
| 60 | 0.08 | -0.29 |
| 61 | 0.08 | -0.30 |
| 62 | 0.08 | -0.30 |
| 63 | 0.08 | -0.30 |
| 64 | 0.08 | -0.29 |
| 65 | 0.08 | -0.30 |
| 66 | 0.08 | -0.30 |
| 67 | 0.08 | -0.29 |
| 68 | 0.08 | -0.30 |
| 69 | 0.08 | -0.29 |
| 70 | 0.08 | -0.30 |
| 71 | 0.08 | -0.30 |
| 72 | 0.08 | -0.29 |
| 73 | 0.08 | -0.30 |
| 74 | 0.08 | -0.30 |
| 75 | 0.08 | -0.30 |
| 76 | 0.08 | -0.29 |
| 77 | 0.08 | -0.30 |
| 78 | 0.08 | -0.30 |
| 79 | 0.08 | -0.30 |
| 80 | 0.08 | -0.30 |
| 81 | 0.08 | -0.29 |
| 82 | 0.08 | -0.30 |
| 83 | 0.08 | -0.29 |
| 84 | 0.08 | -0.30 |
| 85 | 0.08 | -0.29 |
| 86 | 0.08 | -0.30 |
| 87 | 0.08 | -0.30 |
| 88 | 0.08 | -0.30 |
| 89 | 0.08 | -0.30 |
| 90 | 0.08 | -0.29 |
| 91 | 0.08 | -0.29 |
| 92 | 0.08 | -0.29 |
| 93 | 0.08 | -0.29 |
| 94 | 0.08 | -0.29 |
| 95 | 0.08 | -0.30 |
| 96 | 0.08 | -0.29 |
| 97 | 0.08 | -0.29 |
| 98 | 0.08 | -0.29 |
| 99 | 0.08 | -0.30 |
| 100 | 0.08 | -0.29 |
| 101 | 0.08 | -0.30 |
| 102 | 0.08 | -0.30 |
| 103 | 0.08 | -0.30 |
| 104 | 0.08 | -0.30 |
| 105 | 0.08 | -0.30 |
| 106 | 0.08 | -0.30 |
| 107 | 0.08 | -0.30 |
| 108 | 0.08 | -0.30 |
| 109 | 0.08 | -0.30 |
| 110 | 0.08 | -0.30 |
| 111 | 0.08 | -0.30 |
| 112 | 0.08 | -0.30 |
| 113 | 0.08 | -0.30 |
| 114 | 0.08 | -0.29 |
| 115 | 0.08 | -0.29 |
| 116 | 0.08 | -0.29 |
| 117 | 0.08 | -0.32 |
| 118 | 0.08 | -0.30 |
| 119 | 0.08 | -0.29 |
| 120 | 0.08 | -0.30 |
| 121 | 0.08 | -0.30 |
| 122 | 0.08 | -0.30 |
| 123 | 0.08 | -0.30 |
| 124 | 0.08 | -0.30 |
| 125 | 0.08 | -0.29 |
| 126 | 0.08 | -0.29 |
| 127 | 0.08 | -0.30 |
| 128 | 0.08 | -0.30 |
| 129 | 0.08 | -0.29 |
| 130 | 0.08 | -0.29 |
| 131 | 0.08 | -0.29 |
| 132 | 0.08 | -0.29 |
| 133 | 0.08 | -0.29 |
| 134 | 0.08 | -0.30 |
| 135 | 0.08 | -0.29 |
| 136 | 0.08 | -0.29 |
| 137 | 0.08 | -0.29 |
| 138 | 0.08 | -0.29 |
| 139 | 0.08 | -0.29 |
| 140 | 0.08 | -0.30 |
| 141 | 0.08 | -0.29 |
| Avg | 0.1 | -0.3 |
| SD | 0.0 | 0.0 |

AROMO: Aromaticity, Avg: Average, SD: Standard deviation
